# Expression profiling of *Echinococcus multilocularis* miRNAs throughout metacestode development *in vitro*

**DOI:** 10.1371/journal.pntd.0009297

**Published:** 2021-03-22

**Authors:** Natalia Macchiaroli, Matías Preza, Matías Gastón Pérez, Laura Kamenetzky, Marcela Cucher, Uriel Koziol, Estela Castillo, Matthew Berriman, Klaus Brehm, Mara Cecilia Rosenzvit

**Affiliations:** 1 Laboratorio Biología Molecular de Hidatidosis, Instituto de Microbiología y Parasitología Médica (IMPaM), Consejo Nacional de Investigaciones Científicas y Tecnológicas (CONICET), Facultad de Medicina, Universidad de Buenos Aires (UBA), Buenos Aires, Argentina; 2 Laboratorio de Genómica y Bioinformática de Patógenos, Instituto de Biociencias, Biotecnología y Biología Traslacional (iB3), Departamento de Fisiología y Biología Molecular y Celular, Facultad de Ciencias Exactas y Naturales, Universidad de Buenos Aires (UBA), Buenos Aires, Argentina; 3 Sección Biología Celular, Facultad de Ciencias, Universidad de la República, Uruguay; 4 Sección Bioquímica, Facultad de Ciencias, Universidad de la República, Uruguay; 5 Wellcome Sanger Institute, Wellcome Genome Campus, Hinxton, Cambridgeshire, United Kingdom; 6 University of Würzburg, Institute for Hygiene and Microbiology, Consultant Laboratory for Echinococcosis, Würzburg, Germany; Universidad de la Republica, Uruguay, URUGUAY

## Abstract

The neglected zoonotic disease alveolar echinococcosis (AE) is caused by the metacestode stage of the tapeworm parasite *Echinococcus multilocularis*. MicroRNAs (miRNAs) are small non-coding RNAs with a major role in regulating gene expression in key biological processes. We analyzed the expression profile of *E*. *multilocularis* miRNAs throughout metacestode development *in vitro*, determined the spatial expression of miR-71 in metacestodes cultured *in vitro* and predicted miRNA targets. Small cDNA libraries from different samples of *E*. *multilocularis* were sequenced. We confirmed the expression of 37 miRNAs in *E*. *multilocularis* being some of them absent in the host, such as miR-71. We found a few miRNAs highly expressed in all life cycle stages and conditions analyzed, whereas most miRNAs showed very low expression. The most expressed miRNAs were miR-71, miR-9, let-7, miR-10, miR-4989 and miR-1. The high expression of these miRNAs was conserved in other tapeworms, suggesting essential roles in development, survival, or host-parasite interaction. We found highly regulated miRNAs during the different transitions or cultured conditions analyzed, which might suggest a role in the regulation of developmental timing, host-parasite interaction, and/or in maintaining the unique developmental features of each developmental stage or condition. We determined that miR-71 is expressed in germinative cells and in other cell types of the germinal layer in *E*. *multilocularis* metacestodes cultured *in vitro*. MiRNA target prediction of the most highly expressed miRNAs and *in silico* functional analysis suggested conserved and essential roles for these miRNAs in parasite biology. We found relevant targets potentially involved in development, cell growth and death, lifespan regulation, transcription, signal transduction and cell motility. The evolutionary conservation and expression analyses of *E*. *multilocularis* miRNAs throughout metacestode development along with the *in silico* functional analyses of their predicted targets might help to identify selective therapeutic targets for treatment and control of AE.

## Author summary

Alveolar echinococcosis (AE) is a zoonotic disease caused by the metacestode stage of the helminth parasite *Echinococcus multilocularis*. Current treatment requires surgery and/or prolonged drug therapy. Thus, novel strategies for the treatment of AE are needed. MicroRNAs (miRNAs), a class of small ~22-nucleotide (nt) non-coding RNAs with a major role in regulating gene expression, have been suggested as potential therapeutic targets for treatment and control of helminth parasite infections. In this work, we analyzed the expression profile of *E*. *multilocularis* miRNAs throughout metacestode development *in vitro*. We predicted functional roles for highly expressed miRNAs and found that they could be involved in essential roles for survival and development in the host. We determined that *E*. *multilocularis* miR-71, a highly expressed miRNA that is absent in the human host, is expressed in germinative cells and in other cell types of the germinal layer in *E*. *multilocularis* metacestodes cultured *in vitro*. Germinative cells are a relevant cell type to target for anti-echinococcosis drug development. MiRNAs that are absent in the human host, involved in essential functions, highly expressed and/or expressed in germinative cells in *E*. *multilocularis* metacestodes may represent selective therapeutic targets for treatment and control of AE.

## Introduction

The small tapeworms *Echinococcus multilocularis* and *Echinococcus granulosus* are the etiological agents of alveolar echinococcosis (AE) and cystic echinococcosis (CE), respectively. These diseases are among the most severe parasitoses in humans and belong to a group of priority neglected zoonotic diseases identified by the World Health Organization (WHO) (https://www.who.int/neglected_diseases/zoonoses/infections_more/en/).

The indirect life cycle of *E*. *multilocularis* (fox tapeworm) is complex and involves several developmental transitions. Adult worms develop in the small intestine of definitive hosts (mainly foxes) and produce infective eggs containing the oncospheres, which are released with the host faeces into the environment. The infective eggs ingested by the intermediate hosts (small rodents, accidentally humans) release the oncosphere that after penetrating the intestinal epithelium develops into the metacestode larval stage in host internal organs, with almost all primary infections occurring in the liver [1]. The mature metacestode comprises an inner, cellular germinal layer and an outer, glycan-rich and acellular laminated layer. The germinal layer produces protoscoleces, each with an invaginated scolex within a small posterior body that resembles the anterior region of the adult worm. After ingestion by the definitive host, each scolex evaginates, attaches to the intestinal mucosa and develops into an adult worm [2–4].

AE is caused by the metacestode larval stage, which develops in the liver, grows infiltratively like a malignant tumor into the surrounding host tissue and has the ability to form metastases in secondary organs at late stages of the disease [5–7]. The development of the *E*. *multilocularis* metacestode and its continuous growth and proliferation potential are driven by the germinative cells, the only proliferating cells which represent a unique stem cell system in *E*. *multilocularis* metacestodes [3]. Cestode germinative cells are a population of proliferating undifferentiated cells equivalent to the neoblasts of free-living platyhelminths. These cestode cells are heterogeneous at the molecular level, including their expression of classical pluripotency markers [3] and probably comprise truly pluripotent stem cells and their differentiation-committed immediate progeny with reduced pluripotency. Current treatment requires surgery and/or prolonged drug therapy using benzimidazoles, which are parasitostatic rather than parasiticidal [5,6]. Thus, novel strategies for the treatment of AE are urgently needed [1,8]. The importance of targeting germinative cells in anti-echinococcosis drug development has also been previously suggested [1,9].

The cultivation systems for the *in vitro* maintenance of metacestode vesicles and for primary cells have previously been established in *E*. *multilocularis* [1,10,11]. The primary cell culture, which contains a large proportion of germinative cells [3], has been used to mimic the transition of oncosphere-derived germinative cells into metacestode vesicles *in vitro*. These *in vitro* systems allowed for the study of the cellular and molecular basis of host-parasite interaction and developmental processes in *E*. *multilocularis* [12,13], including the role of miRNAs [14]. Given the unique features of *E*. *multilocularis* metacestode development outlined above, we hypothesize that regulation of gene expression at different levels is important in the control of the underlying morphological and physiological changes.

MicroRNAs (miRNAs) are a class of small ~22-nucleotide (nt) non-coding RNAs with a major role in regulating gene expression that complements and expands the regulation occurring at other levels of the gene-expression program [15]. The importance of miRNAs in key biological processes such as development, cell proliferation, cell differentiation and metabolism has been widely documented [16]. In the canonical pathway of miRNA biogenesis in animals, miRNAs are transcribed from the genome as long primary transcripts (pri-miRNAs). The pri-miRNAs form hairpin structures that are processed through consecutive cleavages by two RNase III enzymes. The first cleavage generates a ~70 nt hairpin precursor miRNA (pre-miRNA) that is further processed to generate a ~22 nt miRNA/miRNA* duplex comprising the mature miRNA and its partially complementary strand (star miRNA). Mature miRNAs are incorporated into an effector miRNA Induced Silencing Complex (miRISC) and bind to complementary sequences of target mRNAs, typically located in the 3’ untranslated regions (3’UTRs). This promotes the repression of protein translation and/or destabilization of the target mRNA. The targeting properties mainly depend on the seed region, an evolutionarily conserved sequence located in the 5’ end of the mature miRNA (nucleotides 2–8) [17].

MiRNAs have been identified in many parasitic helminths of medical and veterinary importance, showing developmental expression patterns [18]. In addition, miRNAs have been reported to be released from these organisms. Thus, parasite-derived miRNAs have been proposed as promising biomarkers for the early detection of helminth parasite infections [18–20] and as potential functional molecules in host-parasite interaction [21–25]. In addition, parasite miRNAs have been suggested as potential therapeutic targets for treatment and control of helminth parasite infections [26–28].

In previous work, we analyzed the miRNA expression profile in *E*. *canadensis* [29,30], *E*. *granulosus sensu stricto* (s. s.) [30], *Mesocestoides vogae* [31], *Taenia crassiceps* [32] and *Hymenolepis microstoma* [33]. Regarding *E*. *multilocularis*, we previously described the miRNA repertoire and expression profile in *E*. *multilocularis in vivo* metacestodes [29] and *E*. *multilocularis* primary cell cultures [14]. In this latter study, we also predicted potential targets of miR-71 after knocking down this miRNA. However, current knowledge about miRNA expression and their potential roles throughout *E*. *multilocularis* metacestode development is still limited. The aims of the present study were to analyze the miRNA expression profile throughout metacestode development *in vitro*, to determine the spatial expression of miR-71 in metacestodes cultured *in vitro* and to predict miRNA targets in *E*. *multilocularis*.

## Methods

### Ethics statement

All experiments in animals were carried out in accordance with European and German regulations on the protection of animals (Tierschutzgesetz, Section 6). The local ethics committee from the government of Lower Franconia issued ethical approval of this study (permit no. 55.2–2531.01-61/13).

### Parasite material

*E*. *multilocularis* isolates were propagated and continuously kept in mongolian jirds (*Meriones unguiculatus*) as described previously [11]. Isolation and processing of *in vivo* parasite material, *in vitro* cultivation of metacestode vesicles and isolation of primary cell cultures were carried out according to established protocols [11].

**Fig 1:**
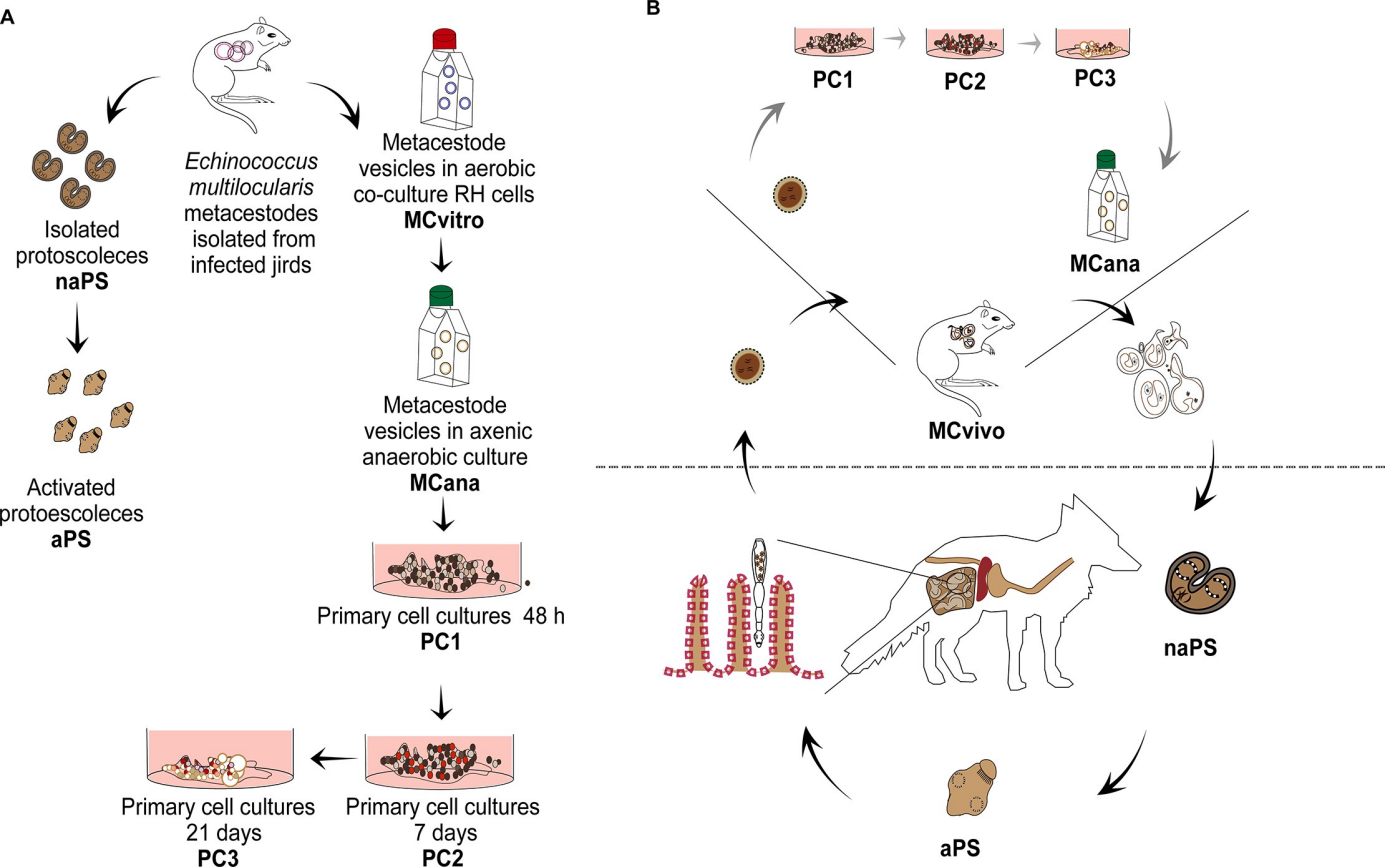
*Echinococcus multilocularis* samples analyzed in this work and their relationship with the stages of the parasite’s life cycle *in vivo*. MCvivo: metacestodes extracted from experimentally infected jirds, MCvitro: metacestodes cultured *in vitro* in aerobic conditions, MCana: metacestodes cultured *in vitro* in axenic and anaerobic conditions, PC1: primary cells cultured *in vitro* for 48 hs, PC2: primary cells cultured *in vitro* for 7 days containing central cavities and PC3: primary cells cultured *in vitro* for 21 days containing several central cavities and released mini vesicles (metacestodes), naPS: non-activated protoscoleces, aPS: *in vitro* activated protoscoleces by pepsin/low pH treatment. *Echinococcus multilocularis* samples analyzed in this work (A). Relationship between *Echinococcus multilocularis* samples analyzed in this work and the stages of *Echinococcus multilocularis* life cycle *in vivo* (B).

Metacestode material was injected intraperitoneally into adult jirds and let develop for 60 days. When established, the metacestodes were dissected from the jirds (MCvivo sample). From this *in vivo* parasite material, protoscoleces were isolated according to an established protocol (naPS sample) [34]. One fraction of the isolated protoscoleces was activated *in vitro* by pepsin/low pH treatment as described previously (aPS sample) [35]. Also, some samples of the *in vivo* parasite material (MCvivo sample) were then co-cultured with RH cells (rat hepatoma cells) in aerobic conditions and harvested when 3–6 month old as described previously (MCvitro sample) [11]. Metacestode vesicles cultured *in vitro* were then moved to axenic and anaerobic conditions for at least three days (MCana sample) [11]. These samples were microscopically inspected to verify the absence of feeder cells. Metacestode vesicles cultured *in vitro* (MCana sample) were used to generate primary cell cultures as previously described [11]. *T*he primary cell cultures were harvested at three different times. *A*fter 48 hs, the cultures formed loose mini-aggregates (PC1 sample). After 7 days, these cells divided or re-aggregated and the cultures formed fused aggregates with a moderate amount of central cavities (PC2 sample). After 21 days, the cultures showed a high amount of central cavities and released mini vesicles (PC3 sample) (Figs 1 and S1).

In order to achieve more standardized samples, the yield of the primary cell isolation process was quantified by measuring the cell density photometrically. Using polystyrene cuvettes of 10 x 4 mm, (Sarstedt, Nümbrecht, Germany) 12.5 μl of the initial cell suspension were diluted with 987.5 μl 1xPBS. The optical density of this dilution was measured at a wavelength of 600 nm employing a Hitachi U-2000 spectrophotometer (New York, USA). The quantity of cell units per μl of cell suspension was calculated by dividing the measured value of the optical density by 0.25. The *in vitro* cultivation of primary cells was carried out in 6-well plates (Nunc, Roskilde, DK) using 600 cell units as starting material and 5.0 ml of medium Ax-cDMEM-A [11] under nitrogen atmosphere. Every 2–3 days 3.0 ml of culture medium was renewed and the cultures monitored using an inverted microscope. All samples were washed three times with ice-cold 1x PBS (phosphate-buffered saline). Metacestode vesicles cultured *in vitro* were pierced with a needle prior to washing, in order to remove hydatid fluid. All samples were transferred to Trizol (Invitrogen, Germany) and stored at -80°C until RNA extraction.

### Small RNA isolation, library construction and sequencing

Small RNA isolation, library construction and sequencing were carried out as previously described [33]. Briefly, samples were mechanically homogenized in 1 ml of Trizol (Invitrogen) for 10 s. Then, 200 μl of chloroform:isoamyl alcohol (24:1) were added and mixed thoroughly. Phase separation was carried out by centrifugation at maximum speed at 4°C. Then, 0.5 volumes of isopropanol and 4 μl of glycogen (5 mg/ml) were added to the aqueous phase and the RNA was pelleted by centrifugation at maximum speed at 4°C for 30 min. The resulting pellet was washed with 70% ethanol, air dried, and resuspended in nuclease-free water. The amount and integrity of total RNA was determined using a 2100 BioAnalyzer (Agilent, USA). RNA was concentrated by ethanol precipitation at -20°C overnight after elimination of polyadenylated mRNA using oligo-dT dynabead. The resulting pellet was re-suspended in 6 μl of nuclease-free water and used as the input material.

Small RNA libraries were prepared using the NEBNext Multiplex Small RNA Library Prep Set for Illumina (NEB, USA). After adapters ligation, reverse transcription followed by PCR enrichment of the libraries were performed. The small RNA libraries were size selected, cleaned up through columns and quantified using the Agilent Bioanalyzer. Small RNA libraries were sequenced using an Illumina sequencing platform (HiSeq 2500) for 100 cycles. For each sample type, three libraries were constructed from three independent samples in order to count with biological replicates. Also, three technical replicates were sequenced for each biological replicate and then pooled for bioinformatic analysis. The small RNAseq data are available in ArrayExpress under accession code E-ERAD-236 (https://www.ebi.ac.uk/arrayexpress/experiments/E-ERAD-236/). Data from primary cells cultured *in vitro* for 48 hs (PC1 samples) recently published by our group [14] were included in the present study for comparative purposes. Although previously published, PC1 samples are part of the same experiment presented in this study and were subjected to the same experimental and bioinformatic workflow as the other samples here included.

### Source of genome assembly and annotations

The ∼115 Mb *E*. *multilocularis* genome assembly (PRJEB122) and the gene annotations of coding genes, tRNAs, rRNAs [36] were downloaded from the WormBase ParaSite database version WBPS14 [37]. MiRNA sequences from all species used for comparative purposes were obtained from miRBase v.22 [38] and from our previous works [14,32,33].

### Small RNA library sequence analysis and classification

Illumina raw sequence reads produced by deep sequencing were preprocessed before mapping to *E*. *multilocularis* genome assembly as previously described [30]. After adapter trimming with cutadapt [39] version 1.18 using default parameters, low quality reads and reads shorter than 18 nt were removed using also cutadapt (—minimum-length 18 -q 33) to obtain clean reads. Then, identical clean reads were collapsed into unique sequences with associated read counts. To classify all small RNA sequences into RNA categories, the processed reads were first mapped to *E*. *multilocularis* genome assembly with Bowtie [40] version 1.2.1 using default parameters except for -v 2 that allows up to two mismatches as previously described [33]. All mapped reads were then BLAST searched [41] version 2.7.1 using these parameters: e-value 0.01, short sequences optimization: -task blastn-short against an in-house database (S2 File). The database included *E*. *multilocularis* rRNAs, tRNAs, snRNAs, snoRNAs retrieved from RNAcentral database release 12 [42], *E*. *multilocularis* mRNAs retrived from WormBase ParaSite database version WBPS13 [37] and miRNAs identified in this study. Reads that matched to snRNAs, snoRNAs or with no match to any category were grouped into “others” category.

### MiRNA identification and evolutionary conservation analyses

To identify conserved and novel miRNAs from the small RNA libraries, the miRDeep2 software package [43] was used. The unique sequences were mapped to the *E*. *multilocularis* genome and used as input for miRNA prediction as previously described for this species and other cestodes [29–33]. The initial miRDeep2 output list of candidate miRNA precursors of each library was manually curated to generate a final high confidence set of miRNAs retaining only candidate precursors with (i) miRDeep2 score ≥ 4; (ii) mature reads in more than one biological sample; (iii) star reads and/or seed conservation; (iv) no match to rRNA, tRNA or mRNA. In addition, mature miRNA sequences were compared by BLAST [41] against an in-house database of previously reported *Echinococcus* miRNA sequences obtained by deep sequencing [14,29,30,44] using the BLAST tool at IMPaM local cluster (https://bioinfo.fmed.uba.ar/). To identify miRNA families within *E*. *multilocularis*, all-against-all pairwise sequence alignments were computed using BLAST [41] and all sequences with the same seed region (mature miRNA nucleotides 2–8) were considered members of the same *E*. *multilocularis* miRNA family.

To analyze conservation of all miRNA families found in *E*. *multilocularis*, mature miRNA sequences were compared with those previously reported in miRBase 22 [38] using SSEARCH (e-value 100) for selected phyla, Cnidaria, Nematoda, Arthropoda, Annelida and the subphylum Vertebrata, using only a seed match criterion (identical nucleotides 2–8 in the 5’end of the mature miRNA). To analyze conservation of *E*. *multilocularis* miRNA sequences across Platyhelminthes, the species used for comparative analysis were selected based on the following criterion: the species with the most complete miRNA complement for a given genus identified by small RNA sequencing. Selected species were the tapeworms *E*. *canadensis* and *E*. *granulosus s*. *s*. [30], *H*. *microstoma* [33], *M*. *vogae* [31], *T*. *crassiceps* [32], the trematode *Schistosoma mansoni* [45–47], the monogenean *Gyrodactylus salaris* [48] and the free-living platyhelminth *Schmidtea mediterranea* [49,50].

### MiRNA genomic location and cluster analyses

The genomic coordinates (scaffold, coordinates, and strand) and location (intronic, exonic and intergenic) of all miRNAs identified in this study were assessed by BLAST searches against current annotations of *E*. *multilocularis* genome available in WormBase ParaSite database version WBPS14 [37] (e-value 0.01). For intronic miRNAs, only miRNAs overlapping a coding gene with a predicted functional annotation and in the same orientation were considered. To identify miRNA clusters in *E*. *multilocularis*, the genomic arrangement of the miRNAs identified in this study was assessed. Precursor miRNA sequences were considered to be grouped in clusters if they were in the same scaffold/contig, less than 10 kb apart and on the same strand [51].

### MiRNA expression profiling and differential expression analysis

Analysis of miRNA expression were performed in R version 4.0.3 (https://cran.r-project.org/). First, normalization of miRNA raw reads in each biological replicate from each sample type was performed using the regularized log transformation function (rlog) from DESeq2 package version 1.30.0 [52]. A matrix of miRNA raw reads from each biological replicate was used as input. Thus, normalization of miRNA raw reads does not take into account the total number of mapped reads in a sample. The output matrix of normalized miRNA read counts was used as input for principal component analysis (PCA), heatmap and correlation analysis. Then, a PCA plot of all biological replicates was performed with DESeq2 package version 1.30.0 [52]. Also, a heatmap including all biological replicates was performed using the pheatmap package version 1.0.12 (https://cran.r-project.org/web/packages/pheatmap/index.html) with default parameters except for cutree_rows = 3 (this option introduces breaks to visualize row clusters). Pearson’s correlation plots between pairs of independent biological replicates from a given sample type were created in R. Finally, differential expression analysis of miRNAs between samples was performed by DESeq2 using raw reads as input [53]. For each comparison, miRNAs expressed in both samples that showed a log2 fold change ≥ 1 and p-adjusted <0.05 were considered differentially expressed.

### Whole mount *in situ* hybridization (WMISH) of miR-71 in metacestodes cultured *in vitro*

The WMISH protocol for detection of miR-71 expression in *E*. *multilocularis* metacestodes cultured *in vitro* (MCvitro sample) was adapted from [3]. MCvitro samples were hybridized with a miR-71 Locked Nucleic Acid (LNA) antisense oligonucleotide (5’-ATCTCACTACCATCGTCTTTCA-3’) (Biomers.net, Germany) or with a scrambled oligonucleotide (5’-GTGTAACACGTCTATACGCCCA-3’) as a negative control. Both oligonucleotides were labeled with digoxigenin (DIG) at the 5’ and 3’ ends and detected with an anti-digoxigenin antibody conjugated to horseradish peroxidase (HRP, Sigma-Aldrich) followed by development with tyramide- FITC. Hybridization was performed at 65°C and the final concentration of the probes was 1 nM. The stain 4′,6-diamidino-2-phenylindole (DAPI) was used as a nuclear stain at a concentration of 1 μg/mL. In some experiments, the thymidine analog 5-ethynyl-2′-deoxyuridine (EdU) (Life Technologies, Darmstadt, Germany) was used as a cell proliferation marker. In these cases, the metacestodes were cultured with 10 μM EdU for five hours before fixation of the material as previously described [3]. For quantifying cells with miR-71 expression and co-localization with EdU, cells were manually counted using Fiji Is Just ImageJ (FIJI) [54].

### MiRNA target prediction and *in silico* functional analysis

MiRNA target prediction was performed for the most expressed miRNAs found in this work (miR-71, miR-9, let-7, miR-1, miR-4989, miR-10) using the *E*. *multilocularis* mature miRNA sequences. Also, a set of predicted three-prime untranslated regions (3’UTRs) was generated for *E*. *multilocularis* based on the integration of RNAseq data with genomic data as previously performed for *E*. *canadensis* [55]. The miRanda algorithm [56] was used to perform the prediction of miRNA target sites with the following parameters: i) strict seed pairing; ii) score threshold: 140; iii) energy threshold: -17 kcal/mol; iv) gap open penalty: -9; v) gap extend penalty: -4; vi) scaling parameter: 4; as previously performed [14,32,55]. In addition, the RNAhybrid algorithm [57] was used for miRNA target prediction with the following parameters: i) energy threshold -20 kcal/mol ii) no G:U in the seed iii) helix constraint 2 to 8 iv) worm p-value. Then, results from both algorithms were integrated and only those targets predicted by both algorithms examined further.

Evolutionarily conservation analysis of miRNA targets was performed in *E*. *granulosus* and *Taenia solium*. Briefly, genomic sequences located downstream the stop codon of orthologous coding genes in *E*. *granulosus* and *T*. *solium* were retrieved from the WormBase ParaSite database version WBPS15 [37] using the BioMart tool [58]. Then, miRNA target prediction was performed in *E*. *granulosus* and *T*. *solium* with miRanda algorithm as described for *E*. *multilocularis*.

In order to perform an *in silico* functional analysis of miRNA targets, functional annotations of *E*. *multilocularis* were retrieved from the WormBase ParaSite database version WBPS14 [37]. In addition, pathway mappings of miRNA targets were obtained from the KEGG database [59] using the BlastKOALA tool [60,61]. In addition, a GO functional enrichment analysis of predicted miRNA targets for each individual miRNA was performed using g:Profiler [62] version e102_eg49_p15_7a9b4d6 with default parameters except for: i) organism: *Echinococcus multilocularis* ii) significance threshold: 0.05 iii) statistical domain scope: custom iv) data sources: GO MF (Molecular Function), GO CC (Cellular Component), GO BP (Biological Process). Only the set of genes used as input for miRNA target prediction was used as a reference background.

## Results and discussion

### Small RNA library sequence analysis and classification

To identify the whole repertoire of miRNAs expressed in *E*. *multilocularis* and compare miRNA expression throughout metacestode development, we sequenced small RNA libraries from three biological replicates of different sample types of *E*. *multilocularis*: metacestodes extracted from experimentally infected jirds (MCvivo), metacestodes cultured *in vitro* in aerobic conditions (MCvitro), metacestodes cultured *in vitro* in anaerobic conditions (MCana), primary cell cultures cultured *in vitro* for 48 hs (PC1), primary cells cultured *in vitro* for 7 days (PC2), primary cells cultured *in vitro* for 21 days (PC3), non-activated protoscoleces (naPS) and activated protoscoleces (aPS) (Figs 1 and S1).

The *in vitro* regeneration system of *E*. *multilocularis* primary cell culture [10] is considered a suitable model to mimic, *in vitro*, the transition of oncosphere-derived stem cells into metacestode vesicles [12,13,63,64]. The primary cell cultures PC1, PC2 and PC3 represent samples at which the germinative cells extracted from metacestodes are starting to re-grow into immature metacestodes. Here we used samples PC1, PC2 and PC3 to emulate the development of immature metacestodes from onchospheres *in vitro*. The MCana sample represents mature metacestodes cultured in the same conditions as PC1, PC2 and PC3 (axenic and anaerobic conditions). MCvivo samples represent metacestodes growing in the intermediate host. After being extracted from experimentally infected jirds, MCvivo samples were co-cultured with rat hepatoma cells under aerobic conditions (MCvitro) and after several months where moved into axenic and anaerobic conditions (Mcana), which is a necessary step to establish the *in vitro* regeneration system. In the intermediate host, mature metacestodes are able to develop protoscoleces, which are dormant (non-activated). Dormant protoscoleces (naPS) become activated in the definitive host by the action of digestive enzymes such as pepsin. We used pepsin/low pH to activate protoscoleces (aPS) [35] (Fig 1).

After trimming and filtering, between ~ 1.5 and 38 million reads per sample were mapped to the high-quality *E*. *multilocularis* genome [36]. We obtained a high percentage of genome mapping in all sample types (>90%), with the only exception of MCvivo samples (~ 57%). The lower proportion of sequences from these samples that mapped to the *E*. *multilocularis* genome was probably due to the presence of host-originating material since *E*. *multilocularis in vivo* metacestodes grow by infiltrating host tissues. A similar proportion of genome mapping for *E*. *multilocularis in vivo* metacestodes (~ 60%) was previously reported [29]. The general results of the Illumina deep sequencing are shown in S1 Table. To determine the composition of the small RNA libraries, all mapped reads were classified into miRNAs, rRNAs, tRNAs, mRNAs, snRNAs and snoRNAs. We obtained a different proportion of miRNA reads, ranging from ~ 4% to ~60%, depending on each biological replicate and sample type (S1 Table).

MiRNAs were the most abundant category of small RNAs in both protoscolex samples naPS and aPS, accounting for ~38% to ~60% of the total mapped reads. The percentages of miRNA reads in both protoscolex samples were higher than in metacestode and primary cells (PC1) samples (S1 Table). This finding is consistent with the fact that protoscolex stage shows higher morphological organization, complexity and specialized cellular territories compared to metacestode samples. In addition, a higher proportion of miRNAs in protoscolex samples (~50%) compared to metacestode samples (30%) was described for *E*. *canadensis* G7 [30]. A higher proportion of miRNAs in more complex developmental stages during larval organogenesis and morphogenesis was reported in *Ascaris* [65].

### MiRNA identification and evolutionary conservation analyses

We confirmed the expression of the whole miRNA repertoire that has previously been identified by our group in *E*. *multilocularis in vivo* metacestodes [29]. *W*e did not find additional conserved or novel *miRNAs expressed in E*. *multilocularis samples*. The whole repertoire in *E*. *multilocularis* is composed of 37 mature miRNAs (Table 1*)*.

**Table 1:** The repertoire of *Echinococcus multilocularis* mature miRNAs identified through metacestode development *in vitro*, their evolutionary origin and conservation.

miRNA name	Mature sequence (5’-3’)^a^	Family	Evolutionary origin^b^	Conservation in selected Phyla^c^	Conservation in selected Platyhelminthes^d^							
					Egr	Eca	Tcr	Mvo	Hmi	Sma	Gsa	Sme
**emu-bantam-3p**	U**GAGAUCG**CGAUUACAGCUGAU	Bantam	P	+/+/+/–/–	+	+	+	+	+	+	+	+
**emu-let-7-5p**	U**GAGGUAG**UGUUUCGAAUGUCU	let-7	B	+/+/+/+/–	+	+	+	+	+	+	+	+
**emu-miR-1-3p**	U**GGAAUGU**UGUGAAGUAUGU	mir-1	B	+/+/+/+/–	+	+	+	+	+	+	+	+
**emu-miR-2a-3p**	A**AUCACAG**CCCUGCUUGGAACC	mir-2	P	+/+/+/–/–	+	+	+	+	+	+	+	+
**emu-miR-2b-3p**	U**AUCACAG**CCCUGCUUGGGACA				+	+	+	+	+	+	+	+
**emu-miR-2c-3p**	__**UCACAG**CCAAUAUUGAUGAA				+	+	+	+	+	+	+	+
**emu-miR-7a-5p**	U**GGAAGAC**UGGUGAUAUGUUGU	mir-7	B	+/+/+/+/–	+	+	+	+	+	+	+	+
**emu-miR-7b-5p**	U**GGAAGAC**UUGUGAUUAGAUUGUU				+	+	+	+	+	+	+	+
**emu-miR-8-3p**	U**AAUACUG**UUCGGUUAGGACGCC	mir-8	B	+/+/+/+/–	+	+	-	-	-	+	+	+
**emu-miR-9-5p**	U**CUUUGGU**UAUCUAGCUGUGUG	mir-9	B	+/+/+/+/–	+	+	+	+	+	-	+	+
**emu-miR-10-5p**	C**ACCCUGU**AGACCCGAGUUUGA	mir-10	E	+/+/+/+/+	+	+	+	+	+	+	+	+
**emu-miR-31-5p**	U**GGCAAGA**UACUGGCGAAGCUGA	mir-31	B	+/+/+/+/–	+	+	+	+	+	+	+	+
**emu-miR-36a-3p**	U**CACCGGG**UAGACAUUCCUUGC	mir-36	P	+/+/+/–/–	+	+	+	+	+	+	+	+
**emu-miR-36b-3p**	U**CACCGGG**UAGUUAUUACGCCU				+	+	+	+	+	+	+	+
**emu-miR-61-3p**	U**GACUAGA**AAGAGCACUCACAUC	mir-61/279	P	+/+/+/–/–	+	+	+	+	+	+	+	+
**emu-miR-71-5p**	U**GAAAGAC**GAUGGUAGUGAGA	mir-71	B	+/+/+/–/–	+	+	+	+	+	+	+	+
**emu-miR-87-3p**	G**UGAGCAA**AGUUUCAGGUGU	mir-87	P	+/+/+/–/–	+	+	+	+	+	-	+	+
**emu-miR-96-5p**	A**UUGGCAC**UUUUGGAAUUGUC	mir-96	B	+/+/+/+/–	+	+	+	+	+	+	+	+
**emu-miR-124a-3p**	U**AAGGCAC**GCGGUGAAUGCCA	mir-124	B	+/+/+/+/–	+	+	+	+	+	+	+	+
**emu-miR-124b-3p**	U**AAGGCAC**GCGGUGAAUACC				+	+	+	+	+	+	+	+
**emu-miR-125-5p**	U**CCCUGAG**ACCCUAGAGUUGUC	mir-125	B	+/+/+/+/–	+	+	+	+	+	+	+	+
**emu-miR-133-3p**	U**UGGUCCC**CAUUAACCAGCCGCC	mir-133	B	+/+/+/+/–	+	+	+	+	+	-	-	+
**emu-miR-153-3p**	U**UGCAUAG**UCUCAUAAGUGCCA	mir-153	B	+/+/+/+/–	+	+	+	+	+	-	+	+
**emu-miR-184-3p**	G**GGACGGA**AGUCUGAAAGGUUU	mir-184	B	+/+/+/+/–	+	+	+	+	+	-	+	+
**emu-miR-190-5p**	A**GAUAUGU**UUGGGUUACUUGGUGCU	mir-190	B	+/+/+/+/–	+	+	+	+	+	+	+	+
**emu-miR-219-5p**	U**GAUUGUC**CAUUCGCAUUUCUUG	mir-219	B	+/-/+/+/–	+	+	+	+	+	+	+	+
**emu-miR-277a-3p**	U**AAAUGCA**UUUUCUGGCCCGUA	mir-277/4989	P	+/+/+/–/–	+	+	+	+	+	+	+	+
**emu-miR-277b-3p**^**e**^	U**AAAUGCA**AAAUAUCUGGUUAUG				+	+	+	+	+	+	+	+
**emu-miR-4989-3p**	A**AAAUGCA**CCAACUAUCUGAGA				+	+	+	+	+	+	+	+
**emu-miR-281-3p**	U**GUCAUGG**AGUUGCUCUCU	mir-281	B	+/+/+/+/–	+	+	+	+	+	+	+	+
**emu-miR-307-3p**	U**CACAACC**UACUUGAUUGAGGGG	mir-307/67	P	+/+/+/–/–	+	+	+	+	+	-	+	+
**emu-miR-745-3p**	U**GCUGCCU**GGUAAGAGCUGUGA	mir-745/22	B	+/–/+/+/–	+	+	+	+	+	+	-	+
**emu-miR-1992-3p**	U**CAGCAGU**UGUACCAUUGAAAU	mir-1992	L	+/–/–/–/–	+	+	+	-	-	-	+	+
**emu-miR-2162-3p**	U**AUUAUGC**AACUUUUCACUCC	mir-2162/1993	P	+/+/+/–/–	+	+	+	+	+	+	+	+
**emu-miR-3479a-3p**	U**AUUGCAC**GUUCUUUCGCCAUC	mir-3479/92	B	+/+/+/+/–/	+	+	+	+	+	+	-	+
**emu-miR-3479b-3p**	G**AUUGCAC**UACCCAUCGCCCAC				+	+	+	+	+	+	-	+
**emu-miR-10293-3p**^**f**^	U**AAUUCGA**GUCAACAGGGUCGUU	mir-10293	Pl	–/–/–/–/–	+	+	+	+	-	+	+	+

^a^ Seed sequences (nt 2–8) are shown in bold.

^b^ P: Protostomia; B: Bilateria, E: Eumetazoa; L: Lophotrochozoa; Pl: Platyhelminthes.

^c^ Annelida/Nematoda/Arthrophoda/Vertebrata/Cnidaria. (Vertebrata is a Subphylum).

^d^ Egr: *Echinococcus granulosus*, Eca: *Echinococcus canadensis*, Tcr: *Taenia crassiceps*, Mvo: *Mesocestoides vogae*, Hmi: *Hymenolepis microstoma*, Sma: *Schistosoma mansoni*, Gsa: *Gyrodactylus salaris*, Sme: *Schmidtea mediterranea*.

^e^ Previously reported as miR-new-2-3p by Cucher et al. (2015).

^f^ Previously reported as miR-new-1-3p by Cucher et al. (2015).

The whole repertoire of mature, star and precursor miRNA sequences are shown in S2 Table. For each mature miRNA sequence, we detected expression of the corresponding star miRNA sequence. This is consistent with the miRNA biogenesis model and provides confidence to the predictions obtained (S2 Table). During miRNA biogenesis, mature miRNAs are processed from the 5’ or from the 3’ arm of the pre-miRNA and then loaded into an effector complex. We found that most mature miRNAs in *E*. *multilocularis* (~70%) were processed from the 3′ arm (Table 1 and S2). This bias was previously reported for *E*. *multilocularis* [29] and other cestodes such as *E*. *canadensis* [30], *M*. *vogae* [31], *T*. *crassiceps* [32] and *H*. *microstoma* [33]. The 3’ bias was previously described in nematodes, fruit fly and plants but not in vertebrates where the major product originates from the 5’ arm [66]. Post-transcriptional addition of non-templated uridyne(s) to the 3′ end of miRNAs can affect activity, biogenesis and turnover of miRNAs [67]. Since 3’ terminal uridylases can modify not only the mature miRNA but also the pre-miRNA at the 3’ position [68], cestode miRNAs that come from the 3’ arm have more chances of being uridylated [29]. MiRNA uridylation was previously described in *E*. *multilocularis* [29] and *M*. *vogae* [31]. However, the role of miRNA uridylation in cestodes remains unknown.

We classified the 37 mature miRNAs expressed in *E*. *multilocularis* samples into 29 miRNA families according to the conservation of their seed regions (mature miRNA nucleotides 2–8) (Table 1). The seed region is the principal determinant of the interaction between miRNA and mRNA target. Therefore, members of the same miRNA family often have at least partially redundant functions [15]. The phylogenetic distribution of *E. multilocularis* miRNA families was based on previous classification of miRNA families [69,70] and was confirmed by homology searches in miRBase v.22.

Regarding their evolutionary origin, we found one eumetazoan-specific miRNA family, 18 bilaterian-specific miRNA families, eight protostomian-specific miRNA families, one lophotrochozoan-specific miRNA familiy and one platyhelminth-specific miRNA family (family mir-10293, seed AAUUCGA). We did not find either cestode-specific miRNA families or *Echinococcus*-specific miRNA families (Table 1). Thus, 28 miRNA families identified in *E*. *multilocularis* were conserved in different Phyla, whereas one miRNA family (*mir-10293)* was conserved only across Platyhelminthes (Table 1).

The number of miRNA families identified in *E*. *multilocularis* that were found conserved in different phyla (28 miRNA families) is similar to that found in other platyhelminths [33]. Considering that 46 evolutionarily conserved miRNAs are expected for a lophotrochozoan organism [48] the reduced number of conserved miRNA families found in *E*. *multilocularis* is consistent with previous knowledge about the common loss of conserved miRNA families in flatworms [48]. A reduced number of miRNA families has been correlated with a lower morphological complexity [71,72]. In addition, a higher loss of conserved miRNA families in parasitic flatworms with respect to the free-living *S*. *mediterranea* that was found may be reflecting their parasitic lifestyle as previously suggested [30,33,44,48]. Also, the loss of deeply conserved miRNA families in tapeworms may be related to additional morphological and metabolic reductions found in tapeworms, including *E*. *multilocularis* [36] as proposed for *Hymenolepis* [33].

Regarding conservation of the mature miRNA repertoire, most of them were conserved within selected platyhelminths (Table 1). The lack of conservation of miR-8 and miR-1992 in some platyhelminths could be due to poorer quality genome assembly and/or annotation since we also failed to find them bioinformatically in their genomes. However, we cannot rule out that these miRNAs could be present in their genomes.

*E*. *multilocularis* miRNAs that are absent in the host or highly divergent from their host orthologs, such as miR-71 and miR-4989, may provide selective therapeutic targets for treatment and control of echinococcosis.

### MiRNA genomic location and cluster analyses

We determined the genomic coordinates and location of all miRNA precursors identified in *E*. *multilocularis* according to current genome assembly and annotations from the WormBase ParaSite database (S2 Table**)**. Precursor miRNAs mapped to all of the nine *E*. *multilocularis* chromosomes [36]. Three miRNA precursors, mir-190, mir-96 and mir-3479b, were located in introns of protein coding genes (with a predicted functional annotation and in same orientation), whereas most precursor miRNAs, 92% (34/37), were located in intergenic regions distant from annotated genes (S2 Table). This bias was previously reported for *E*. *multilocularis* [29] and was observed in other parasitic platyhelminths such as *Hymenolepis* [33] and *Schistosoma japonicum* [73] where 92% and 90% of the miRNA complement, respectively, was located in intergenic regions. Recently, the three intronic miRNAs identified in *E*. *multilocularis* (mir-190, mir-96 and mir-3479b) were also found to be located in introns of the same protein coding genes in *Hymenolepis and* a comparative analysis of their genomic location and the structure of their host genes were performed across selected platyhelminths with available genomes including *E*. *multilocularis* [33].

We re-assessed the presence of *E*. *multilocularis* miRNA clusters by analyzing the genomic arrangement of the 37 miRNAs identified in this study according to current genome assembly and annotations from the WormBase ParaSite database. Here we confirmed the presence of the two miRNA clusters mir-71/2b/2c and mir-277a/4989 in *E*. *multilocularis* that have previously been identified by our group [29,74]. Each miRNA cluster comprised a genomic region of up to 285 bp, suggesting co-expression as a single transcriptional unit [16] and were located in intergenic regions (S3 Table). The clustering of mir-71 with members of the mir-2 family has also been identified in nematodes [75], suggesting strong evolutionary pressure to maintain this organization. Here, we found a similar percentage of miRNAs in clusters in *E*. *multilocularis* genome ~ 14% (5/37) as previously found in the genomes of *M. vogae* ~ 12% (5/42) [31], *Taenia solium* ~ 18% (7/39) [32] and *Hymenolepis* ~ 20% (8/37) [33].

### MiRNA expression profiling

**Fig 2:**
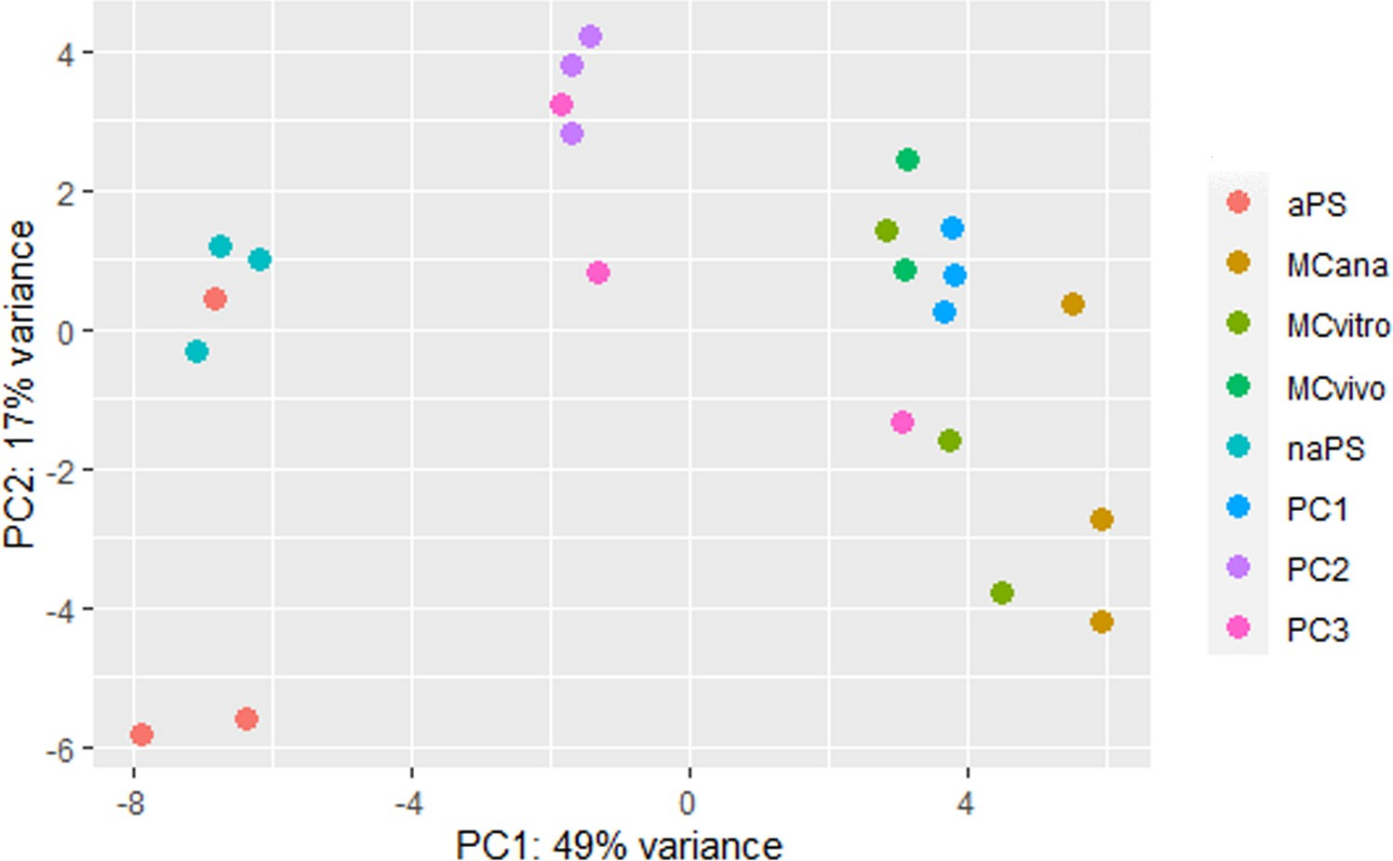
Principal component analysis of the miRNA expression data from different *Echinococcus multilocularis* samples. PCA plot of all biological replicates from different *E*. *multilocularis* samples based on normalized miRNA read counts. Each dot indicates one biological replicate from a given sample. MCvivo: metacestodes extracted from experimentally infected jirds, MCvitro: metacestodes cultured *in vitro* in aerobic conditions, MCana: metacestodes cultured *in vitro* in axenic and anaerobic conditions, naPS: non-activated protoscoleces, aPS: activated protoscoleces, PC1: primary cell cultured *in vitro* for 48 hs, PC2: primary cells cultured *in vitro* for 7 days and PC3: primary cells cultured *in vitro* for 21 days.

To explore biological variation among replicates from different *E*. *multilocularis* samples and to visualize clusters of samples based on their expression profiles, a principal component analysis (PCA) plot of all biological replicates was performed (Fig 2). Previously, normalization of miRNA raw reads in each biological replicate from each sample type was performed (S4 Table).

The plot showed that all biological replicates from naPS, PC2, MCvivo and PC1 samples clustered together, whereas biological replicates from aPS, PC3, MCvitro and MCana samples showed large variation. One biological replicate from aPS samples (aPS_1) clustered with replicates from naPS sample probably due to a failure in the protoscoleces activation protocol (S2 Fig). Also, one biological replicate from MCvitro samples clustered with replicates from MCvivo samples probably due to the heterogeneity of MCvitro replicates. In addition, biological replicates from PC2 and PC3 samples clustered together, with the exception of one replicate from PC3. Although PC2 and PC3 samples were harvested at different number of days (7 and 21 days, respectively) the similarity observed between both samples types may be due to a truly similar miRNA expression pattern since both samples represent immature metacestodes.

The plot showed that the first component clearly splits aPS and naPS samples from the remaining samples. Also, it differentiates PC2 and PC3 samples from MCvivo, MCvitro and MCana and PC1 samples. In contrast, this component showed less differentiation among MCvivo, MCvitro and MCana and PC1 samples. The second component clearly splits aPS from naPS samples (if aPS_1 is excluded from the plot). This analysis showed that the clustering of samples was as expected based on the known morphological, developmental and physiological variation between different samples (Fig 2).

**Fig 3:**
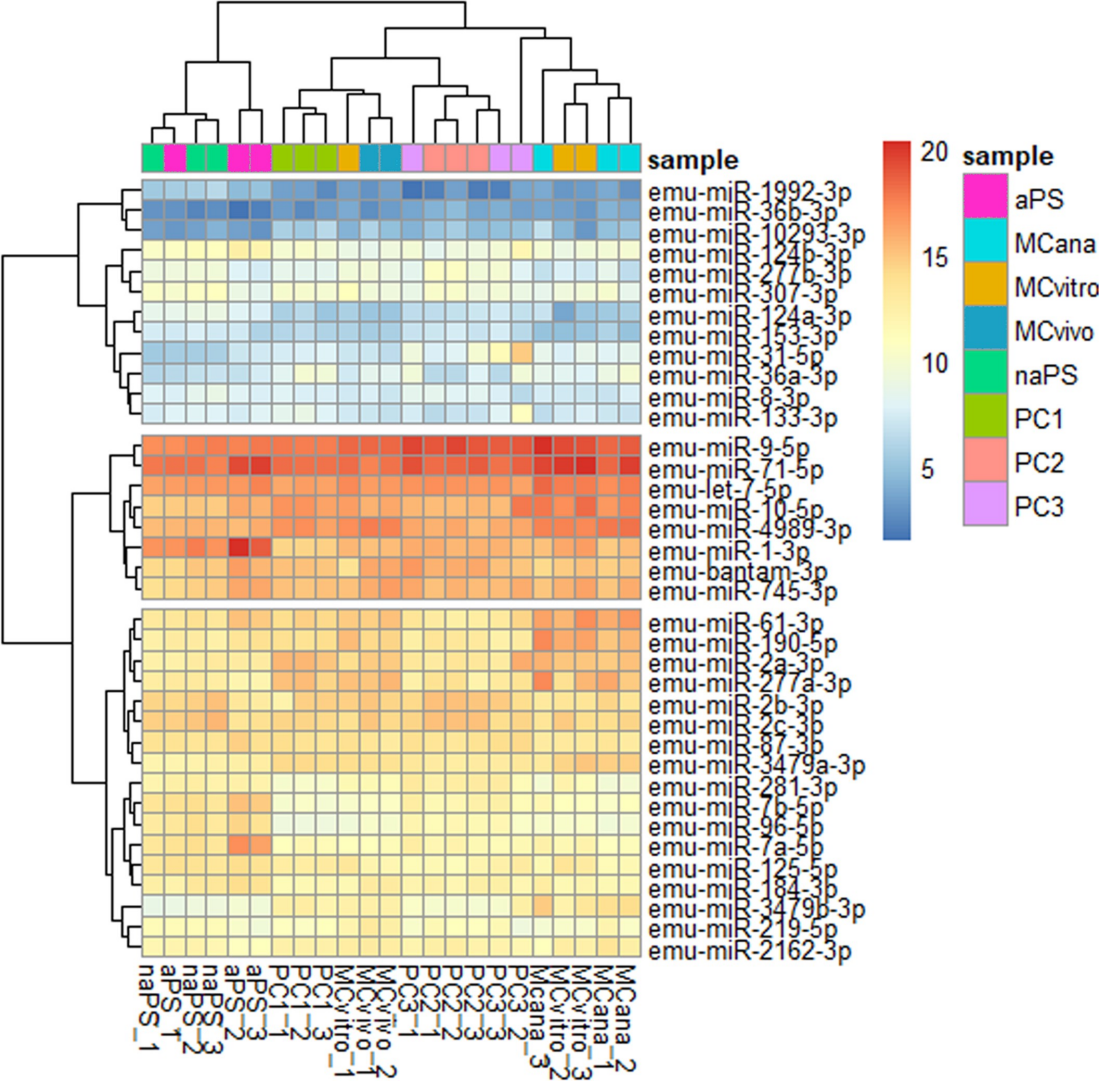
Expression profile of the full miRNA repertoire of *Echinococcus multilocularis* samples. Heatmap of all biological replicates from different *E*. *multilocularis* samples based on normalized miRNA read counts. MiRNA expression level is displayed using a color key where red represents miRNAs with high expression and blue indicates miRNAs with low expression. Hierarchical clustering at a sample level is shown. Three miRNA clusters are also indicated with breaks. MCvivo: metacestodes extracted from experimentally infected jirds, MCvitro: metacestodes cultured *in vitro* in aerobic conditions, MCana: metacestodes cultured *in vitro* in axenic and anaerobic conditions, naPS: non-activated protoscoleces, aPS: activated protoscoleces, PC1: primary cell cultured *in vitro* for 48 hs, PC2: primary cells cultured *in vitro* for 7 days and PC3: primary cells cultured *in vitro* for 21 days.

To visualize the expression profiles of *E*. *multilocularis* mature miRNA repertoire across biological replicates a heatmap was performed (Fig 3). Previously, normalization of miRNA raw reads in each biological replicate from each sample type was performed (S4 Table).

The inclusion of all biological replicates and their hierarchical clustering in the heatmap showed the biological variation found across replicates and samples. Consistent with the principal component analysis plot, the heatmap also showed the incorrect clustering of aPS_1 with naPS samples.

Regarding the global miRNA expression profile, a few miRNAs showed very high expression levels in all *E*. *multilocularis* sample types, whereas most miRNAs showed very low expression (Fig 3 and S4 Table). In this study, we provide for the first time experimental evidence of miRNA expression in *E*. *multilocularis* MCvitro, MCana, naPS, aPS, PC2 and PC3 samples. Previously, we described miRNA expression profiles in *E*. *multilocularis in vivo* metacestodes (MCvivo) [29] and *E*. *multilocularis* primary cells (PC1) [14]. This analysis revealed a strong bias in miRNA expression. Six miRNAs miR-71, miR-9, let-7, miR-10, miR-4989 and miR-1 were the most highly expressed miRNAs across different biological replicates from different *E*. *multilocularis* samples analyzed (Fig 3 and S4 Table). The expression patterns of the miRNA repertoire found in *E*. *multilocularis* samples is overall conserved in closely related species such as *E. canadensis [30], E. granulosus s. s. [30,44], M. vogae* [31] and *T. cracisseps [32]*, suggesting evolutionary and functional importance of *E*. *multilocularis* miRNAs. Although expression patterns of conserved miRNAs are overall conserved over large evolutionary distances, orthologous miRNAs in closely related species can vary in their spatio-temporal expression patterns [72]. In addition, the strong bias in miRNA expression observed in *E*. *multilocularis* and other cestodes was also reported for the trematode *S*. *japonicum*. In this parasite, five miRNAs, including miR-71 and miR-1, where highly expressed throughout life cycle stages [76].

Two of the most expressed miRNAs in all sample types were miR-71 and miR-9. Consistent with these results, miR-71 and miR-9 were previously reported to be highly expressed in *E*. *multilocularis in vivo* metacestodes [29] and *E*. *canadensis* metacestodes [29,30]. Recently, we reported that the knock down of miR-71 in primary cells resulted in the inhibition of metacestode vesicle formation, suggesting that miR-71 is involved in early development in *E*. *multilocularis* [14]. In addition, miR-71 is known to be involved in resistance to heat and oxidative stress, promotion of longevity [77–79] and control of stochastic L/R neuronal asymmetry [80] in *C*. *elegans*. The other highly expressed miRNA, miR-9, is a deeply conserved miRNA across evolution and is known to be involved in neural development [81]. The high expression of miR-71 and miR-9 in all samples suggests that both miRNAs may be essential for *E*. *multilocularis* survival, development and/or host parasite-interaction.

Other highly expressed miRNA in all sample types was let-7, a bilaterian miRNA that is highly divergent at the nucleotide level even among platyhelminths [30]. Let-7 was one of the first discovered miRNAs and is known to be essential for temporal development in *C*. *elegans* [82].

In addition, we found that miR-10 was among the most expressed miRNAs in MCvitro, MCana and PC1 samples. This ancient miRNA is known to have a role in Hox gene regulation [83] [84]. Recently, we found that one of the neighboring genes of mir-10 was a Hox gene as in most bilaterial species [33].

Another highly expressed miRNA in metacestodes samples, MCvivo, MCvitro and MCana, was miR-4989. This miRNA was also highly expressed in primary cells samples, PC1. MiR-4989 (miR-277 family) was found to be highly expressed in *E*. *multilocularis in vivo* metacestodes [29], *E*. *multilocularis* primary cells PC1 [14] and *E*. *canadensis* metacestodes [30]. MiR-4989, is a protostome-specific miRNA that is organized in a cluster with miR-277a, a miRNA known to be involved in amino acid catabolism in *Drosophila* spp. [85]. In addition, it was suggested to be involved in transcriptional regulation during development of juvenile worms of *S*. *mansoni* [47].

One of the most expressed miRNAs in protoscoleces samples, naPS and PS, was miR-1. This miRNA was found to be highly expressed in protoescoleces samples from *E*. *canadensis* and *E*. *granulosus* s. s. [30]. MiR-1 is deeply conserved through evolution with known roles in muscle development [86]. The high expression of this miRNA found only in protoescoleces samples is expected due to a higher level of organization and complexity of muscular system in this developmental stage with respect to metacestodes or primary cells samples from *E*. *multilocularis* [3,87] and in *E*. *granulosus* s. s. [88].

Regarding the expression profile of miRNAs arranged in clusters *mir-71/2b/2c and mir-277a/4989*, we found different levels of miRNA expression between miR-71 and miR-2b/miR-2c and between miR-4989 and miR-277a (S4 Table). We found that miR-71 expression was higher than miR-2b and miR-2c. In addition, miR-4989 expression was higher than miR-277a. Considering that miRNAs in clusters are comprised within a genomic region of up to 285 bp and probably co-expressed in the same cells or tissues as a single transcriptional unit, this result suggest a post-transcriptional regulation of their processing or stability in *E*. *multilocularis*. This result was previously observed in *E*. *multilocularis in vivo* metacestodes [29], *M*. *vogae* [31] and *T*. *crassiceps* [32]. In addition, evidence for post-transcriptional regulation of clustered microRNAs was reported for *Drosophila* [89].

### MiRNA differential expression analysis

To evaluate the variation among biological replicates from different *E*. *multilocularis* samples, we performed a correlation analysis of miRNA expression. Previously, normalization of miRNA raw reads in each biological replicate from each sample type was performed (S4 Table).

We obtained high correlation coefficients (r>0.96), with the only exception of PC3_2 (S3 Fig). For differential expression analysis, biological replicates from PC2 and PC3 samples were grouped (with the exception of PC3_2) and re-named PC2-3 due to their high level of correlation between biological replicates from both sample types (r>0.97) (S4 Fig). In addition, as we previously showed, biological replicates from PC2 and PC3 samples clustered together (Figs 2 and 3).

Although the biological replicate aPS_1 showed a high level of correlation, this replicate was excluded for differential expression analysis due to incorrect clustering (Figs 2 and 3)

To analyze *E*. *multilocularis* miRNA expression throughout metacestode development *in vitro* we performed differential expression analyses of all identified miRNAs between different sample types representing the following *in vivo* transitions or *in vitro* cultured conditions: i) metacestode development from primary cells (PC1 to PC2-3), ii) mature metacestode development from primary cells (PC2-3 to MCana), iii) primary cells isolated from mature metacestodes (PC1 vs MCana), iv) protoscolex development from metacestodes (MCvitro to naPS), v) host-parasite interaction (MCana vs MCvivo), vi) metacestode metabolism (MCvitro to MCana) and vii) activation of protoscoleces (naPS to aPS).

**Fig 4:**
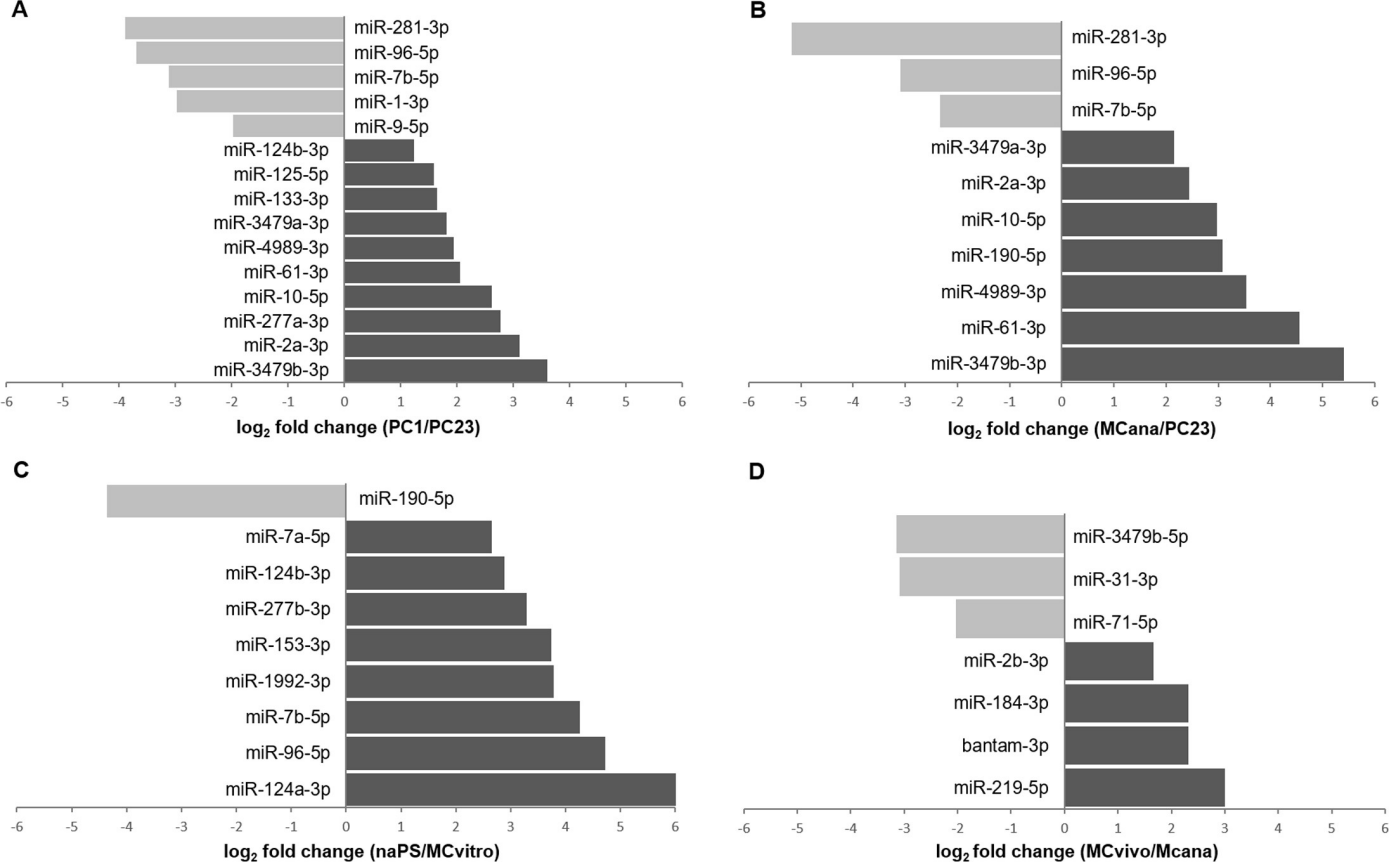
Differential expression analyses of all identified miRNAs between sample types representing different *in vivo* transitions or *in vitro* cultured conditions. MiRNAs that were expressed in both stages but showed log2 fold change ≥1 and p-adjusted <0.05 are shown. PC1 vs PC2-3 (A). MCana vs PC2-3 (B). naPS vs MCvitro (C). MCvivo vs MCana (D). MCvivo: metacestodes extracted from experimentally infected jirds, MCvitro: metacestodes cultured *in vitro* in aerobic conditions, MCana: metacestodes cultured *in vitro* in axenic and anaerobic conditions, PC1: primary cell cultured *in vitro* for 48 hs, PC2-3: primary cells cultured *in vitro* for 7–21 days (see methods section), naPS: non-activated protoscoleces and aPS: activated protoscoleces.

We found significant differences on miRNA expression for the following comparisons: PC1 to PC2-3, PC2-3 to MCana, MCvitro to naPS and MCana vs MCvivo (Fig 4).

#### Metacestode development from primary cells (PC1 to PC2-3)

We found 15 miRNAs differentially expressed between PC1 and PC2-3 (Fig 4A), suggesting that miRNAs may be involved in the early development of metacestode vesicles from primary cells. Ten miRNAs were up-regulated in PC1, a developmental stage enriched in germinative cells. Among them were miR-2a and miR-125, two miRNAs that were found to be enriched in planarian neoblasts [90]. The most up-regulated miRNA in PC1 samples was miR-3479b, a miRNA that is located within the last intron of the conserved mini-chromosome maintenance complex component 2 (mcm2) protein family of DNA helicases in *E*. *multilocularis* [33]. Another member of the same miRNA family, miR-3479a, was also up-regulated in PC1 samples. Two other miRNAs up-regulated in PC1 samples that belong to the same miRNA family as each other and may share a set of mRNA targets were miR-277a and miR-4989. Another up-regulated miRNA in PC1 samples was miR-10, a highly conserved miRNA across metazoans with known roles in development [83,84]. Five miRNAs were up-regulated in PC2-3 samples, including miR-1 that is involved in muscle development [86]. Higher expression of miR-1 was expected in metacestode formation from primary cells since a muscle system is present in *E*. *multilocularis* metacestode cyst wall [3,87,91,92].

#### Mature metacestode development (PC2-3 to MCana)

We found ten miRNAs differentially expressed between PC2-3 and MCana (Fig 4B), suggesting that miRNAs may be involved in the maturation of metacestodes from primary cells. Three miRNAs were up-regulated in PC2-3 samples. These three miRNAs were also up-regulated in PC2-3 samples when compared to PC1. The most up-regulated miRNA was miR-281. This miRNA was found to be involved in developmental transitions in *Bombyx mori* [93]. Another up-regulated miRNA in PC2-3 samples was miR-7b, a miRNA that was found to be enriched in planarian neoblasts [90]. Seven miRNAs were up-regulated in MCana samples. All of them were up-regulated in PC1 samples when compared to PC2-3.

#### Protoscolex development from metacestode (MCvitro to naPS)

We found nine miRNAs differentially expressed between MCvitro and naPS (Fig 4C), suggesting that miRNAs could be important for maintaining particular features of both metacestodes and protoscoleces. Only one miRNA, miR-190, was up-regulated in MCvitro samples. Eight miRNAs were up-regulated in naPS samples, a stage showing a higher morphological organization and complexity. Five of them, miR-124a, miR-124b, miR-96, miR-7a and miR-7b a were also found to be up-regulated in protoscoleces samples compared to metacestodes samples in *E*. *canadensis* [30]. Interestingly, miR-124 is known for its roles in vertebrate neural development [94]. Although nervous system is present in both metacestode larva and protoscolex in *E*. *multilocularis*, it shows further complexity and similarity to that of the adult worm in the protoscolex stage [87].

#### Host-parasite interaction (MCana vs MCvivo)

We found seven miRNAs differentially expressed between MCana and MCvivo (Fig 4D). Since the MCana sample is free from host cells or tissue these miRNAs may be involved in host-parasite interaction. Three miRNAs were up-regulated in MCana samples. Among them was miR-71, a bilaterian miRNA absent in vertebrates. In agreement with this finding, we recently predicted the T-cell immunomodulatory protein (EmTIP) as a potential target of miR-71 and validated their inverse expression [14]. The higher level of miR-71 in MCana samples found here might result in the downregulation of EmTIP, a protein coding gene associated with host-parasite interaction [64], and therefore, expected to be up-regulated in MCvivo samples. Additional miR-71 targets related with host-parasite interaction were predicted in the present study (see MiRNA target prediction and *in silico* functional analysis section). Among them were a tetraspanin [95] and Merlin:moesin:ezrin:radixin, also known as Em2 antigen [96]. Interestingly, miR-71 and miR-3479 were reported to be secreted *in vitro* from the trematode *S*. *mansoni*. Also, miR-71 was detected in sera of *S*. *mansoni* infected mice [22]. In addition, miR-71 was found to be among the most abundant miRNAs found in extracellular vesicles from several nematodes and trematodes species recently analyzed [24]. Also, miR-71 was reported to be secreted *in vitro* from *Taenia crassiceps* [21] and very recently from *E*. *multilocularis* metacestodes [25], suggesting that this miRNA may play a role in host-parasite interaction. Four miRNAs were up-regulated in MCvivo samples. Among them was bantam. Recently, it was demonstrated that bantam regulates host macrophage functions facilitating parasite survival in *S*. *japonicum* [97]. Considering that the *E*. *multilocularis* metacestode lives long term in their host, it would be interesting to determine whether bantam is also involved in modulating the host immune response. However, more experiments are needed in order to determine whether this and the other differentially expressed miRNAs might play a role in host-parasite interaction.

The high proportion of differentially expressed miRNAs found between sample types representing different *in vivo* transitions or *in vitro* cultured conditions suggests that post-transcriptional regulation of particular sets of mRNA targets may play an important role in the *E*. *multilocularis* metacestode development and maintenance of specific features in each developmental stage or *in vitro* cultured condition. This result agrees with the differential expression of protein coding genes found between different sample types in *E*. *multilocularis* [14,36]. However, the importance of the differentially expressed miRNAs in stage transitions and host-parasite interaction should be confirmed by further experimental approaches. In this study, no significant differences were found on miRNA expression between i) PC1 and MCana, ii) MCvitro and MCana and iii) naPS and aPS. These results suggests that miRNAs i) may not differ between primary cells and the mature metacestodes from which they were isolated ii) may not participate in the metacestode metabolism *in vitro* iii) may not participate during protoscoleces activation. We hypothesize that other gene regulation processes, such as transcription regulation by transcription factors, may drive these *in vivo* stage transitions or *in vitro* cultured conditions that were compared.

### Whole mount *in situ* hybridization (WMISH) of miR-71 in metacestodes cultured *in vitro*

**Fig 5:**
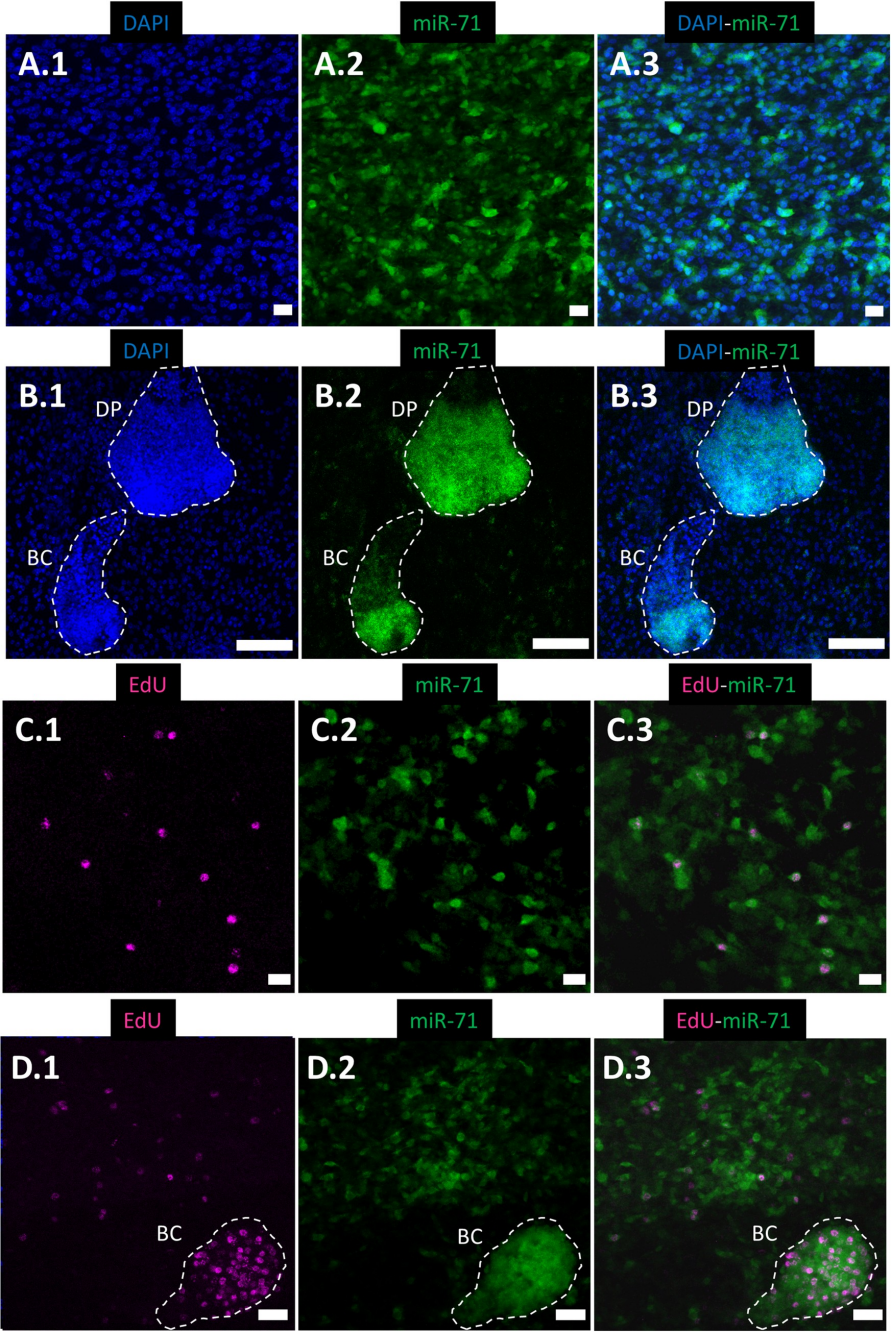
Whole-mount in situ hybridization (WMISH) of miR-71 in *Echinococcus multilocularis* metacestodes cultured *in vitro* (MCvitro). Maximum intensity projection of (A) and (C) germinal layer of *E*. *multilocularis* metacestodes; (B) and (D) germinal layer containing brood capsules (BC) and developing protoscoleces (DP). Nuclear labeling with DAPI (A.1 and B.1, blue); WMISH signal of miR-71 (A.2, B.2, C.2 and D.2, green); detection of proliferation marker EdU (C.1, D.1, magenta); merge of nuclear labeling with DAPI and WMISH signal of miR-71 (A.3 and B.3); and merge of detection of proliferation marker EdU and WMISH signal of miR-71 (C.3 and D.3). Dotted lines delimit BC and DP structures. Scale bar of 10 μm in A, C; 50 μm in B; 20 μm in D.

Detection of individual miRNAs by *in situ* hybridization aids in elucidating miRNA roles in molecular and biological processes [98]. This method has previously been used for detecting miRNA spatial expression in *E*. *multilocularis* protoscoleces [99] and in *Schistosoma* adult worms [47,100]. In this work, we used whole mount *in situ* hybridization (WMISH) to determine the cell-specific expression of miR-71 in *E*. *multilocularis* metacestodes cultured *in vitro* (MCvitro) (Fig 5). This miRNA was the most highly expressed miRNA in MCvitro samples and in primary cell cultures (PC1), a sample type enriched in germinative cells (S4 Table). We hypothesized that miR-71 is expressed in germinative cells, which are proliferating undifferentiated cells equivalent to the neoblasts of free-living platyhelminths and the only proliferating cells of the germinal layer.

We detected miR-71 expression throughout the germinal layer in ~16% of total cells (n = 1615) of *E*. *multilocularis in vitro* metacestodes (Fig 5A.3). No fluorescence signal was observed when the metacestode vesicles were incubated with a scrambled oligonucleotide used as a negative control (S5 Fig). Regarding the level of miR-71 expression, we found a strong signal in brood capsules in different stages of development and in developing protoscoleces (Fig 5B.2 and 5D.2). This result suggests a role of miR-71 in brood capsule and protoscolex development in *E*. *multilocularis* metacestodes cultured *in vitro*.

In a previous study [3], a sample consisting of several *E*. *multilocularis* cell types present in metacestode vesicles was incubated *in vitro* for a short period of time (5 hours) with the thymidine analog EdU. The germinative cells were the only cells that incorporated the proliferation marker, whereas no other morphologically differentiated cells, such as tegumental cells, glycogen/lipid storage cells and calcareous corpuscle cells did. This result indicated that EdU incorporation is a mark of germinative cells and that these cells are the only cells able to proliferate. Here, in order to determine whether miR-71 is expressed in germinative cells, *E*. *multilocularis in vitro* metacestodes were incubated with EdU before performing WISH. We found that several cells that showed EdU incorporation also showed miR-71 expression, confirming the hypothesis that miR-71 is expressed in germinative cells of *E*. *multilocularis in vitro* metacestodes (Fig 5C.3 and 5D.3). This result agrees with previous work showing expression of miR-71b in proliferating neoblasts of the free-living platyhelminth *S*. *mediterranea* [49,50,90]. Although the importance of miRNAs in stem cell pluripotency, reprogramming and differentiation is well documented in mammals [101], their role in stem cell maintenance and differentiation in platyhelminths remains largely unknown. Our results suggest a role of miR-71 in the regulation of gene expression in germinative; a cell population that probably comprise truly pluripotent stem cells and their differentiation-committed immediate progeny with reduced pluripotency, in *E*. *multilocularis* metacestodes cultured *in vitro*. However, we also found cells with EdU signal that did not show miR-71 expression (S1 File). We found that the percentage of cells expressing miR-71 (17.5%) is less than the total amount of germinative cells in metacestodes, which is around 25% [3], suggesting that miR-71 is expressed in several germinative cells but not in all of them. This agrees with a previous report that showed that *E*. *multilocularis* germinative cells are heterogeneous at the molecular level and proposed the existence of germinative cell sub-populations [3]. Since targeting germinative cells has been proposed for anti-echinococcosis drug development [9], miR-71 may be considered a potential therapeutic target for treatment and control of echinococcosis.

In addition, we found cells that showed miR-71 expression but without EdU signal (Fig 5C.3 and 5D.3). Although some may be germinative cells that are not in S phase or progeny cells that directly result from stem cell mitosis, this finding suggests that other cell types, not germinative cells, express miR-71 and therefore, may indicate functional roles other than development and regulation of stem cell pluripotency in *E*. *multilocularis* metacestodes. A broader role of miR-71 is supported by its high expression in all samples analyzed, including those that are not enriched in germinative cells, and by it having the highest number of predicted targets among the six miRNAs analyzed (see MiRNA target prediction and *in silico* functional analysis section). Analyses of predicted functions of miR-71 targets performed in our previous work [14] suggest the importance of this miRNA as a potential therapeutic target for treatment and control of echinococcosis. This agrees with other works that propose targeting miRNAs for treatment and control of helminth parasite infections [26–28].

### MiRNA target prediction and *in silico* functional analysis

To analyze the potential functional roles of the most highly expressed miRNAs in *E*. *multilocularis* samples we performed a miRNA target prediction using miRanda algorithm (S5 Table). We found 309 miRNA target sites between the set of six miRNAs (miR-10, miR-1, miR-9, let-7, miR-4989 and miR-71) and the set of 6,227 *E*. *multilocularis* 3’UTRs used as input for miRNA target prediction. The number of 3’UTRs represents ~58.4% of the total number of coding genes in *Echinococcus multilocularis* (6,227/10,663). Although this number represents only more than a half of the total number of 3’UTRs (and not the total number of coding genes), they are a confident set of 3’UTRs annotated based on the integration of RNAseq data and genomic data. A similar approach for miRNA target prediction using only a subset of 3’UTRs of the total number of coding genes was recently performed in *E*. *canadensis* [55] and in *S*. *mansoni* [47]. These 309 miRNA target sites were located in 303 *E*. *multilocularis* unique target genes, since six target genes: EmuJ_000109300, EmuJ_000484900, EmuJ_000778300, EmuJ_000808600, EmuJ_000189200 and EmuJ_000935200 had two sites for the same or different miRNAs. We found that each miRNA had a different number of predicted target genes. The number of unique targets per miRNA ranged from 11 to 165 depending on the miRNA. MiR-71 was the miRNA with the highest number of targets ~55% (165/303), whereas miR-4989 was the miRNA with the lowest number of targets ~3.6% (11/303) (S5 Table). The high number of targets for miR-71 was previously reported in the closely related species *E*. *canadensis* [55] and in the more distant *S*. *japonicum* [45].

We also performed a miRNA target prediction using RNAhybrid algorithm. We found 420 miRNA target sites between the set of six miRNAs (miR-10, miR-1, miR-9, let-7, miR-4989 and miR-71) and the set of 6,227 *E*. *multilocularis* 3’UTRs used as input for miRNA target prediction (S6 Table). Considering that 275 miRNA target sites were predicted by both miRanda and RNAhybrid algorithms, the integration of the outputs from both algorithms showed very consistent results giving more confidence to the miRNA target predictions obtained.

We determined the evolutionary conservation of the 275 miRNA target sites predicted in *E*. *multilocularis* by both algorithms across the closely related species *E*. *granulosus* and *T*. *solium*. We found that ~75% (206/275) of the miRNA target sites were conserved in at least one species adding weight to the miRNA target predictions obtained (S7 Table). As expected, a higher percentage of conserved miRNA target sites was observed for *E*. *granulosus* ~ 70% (193/275) than for *T*. *solium* ~ 32% (89/275) (S7 Table).

In order to strengthen the miRNA target prediction functional information, we performed a GO functional enrichment analysis of predicted miRNA targets for each individual miRNA. We did not find significant enriched GO terms for any miRNA (S8 Table). We hypothesize that this result might be related to the low number of miRNA targets of each individual miRNA used as input for the enrichment analysis given that many *E*. *multilocularis* coding genes have no annotation and/or associated GO terms [102] and the stringent parameters used for miRNA target prediction.

We also carried out a KEGG pathway analysis and assigned the predicted miRNA target genes to KEGG pathways (S9 Table).

Finally, we selected relevant targets for each miRNA based on the following criteria: i) prediction with both algorithms ii) evolutionary conservation in at least one species (*E*. *granulosus* or *T*. *solium*) iii) assigned gene function iv) potentially related to a known function for the same miRNA in other organisms (Tables 2 and S7).

**Table 2:** Selected target genes of the most expressed miRNAs in *Echinococcus multilocularis*.

miRNA	Gene ID	Gene Description ^a^	KEGG pathway	Conservation
**emu-miR-10-5p**	EmuJ_000389600	Mitogen activated protein kinase kinase kinase	MAPK signaling pathway	*E*. *granulosus* and *T*. *solium*
	EmuJ_000188500	Protein pangolin J	Wnt signaling pathway	*E*. *granulosus* and *T*. *solium*
	EmuJ_001095100	Homeobox protein Meis1	--	*E*. *granulosus* and *T*. *solium*
**emu-miR-1-3p**	EmuJ_000485800	Ezrin-like protein (Elp)	Regulation of actin cytoskeleton	*T*. *solium*
	EmuJ_001049900	Dynein light chain	Cytoskeleton proteins	*E*. *granulosus*
	EmuJ_000596700	Dynein heavy chain	Cytoskeleton proteins	*E*. *granulosus*
	EmuJ_000178100	Activin; Tgf beta family	TGF-beta signaling pathway	*E*. *granulosus*
**emu-miR-9-5p**	EmuJ_000108300	Carbonic anhydrase	--	*E*. *granulosus*
	EmuJ_000955500	Peregrin	Chromosome and associated proteins	*E*. *granulosus*
**emu-miR-4989-3p**	EmuJ_000495100	General transcription factor IIH subunit	Basal transcription factors	*E*. *granulosus*
	EmuJ_001177300	Basic leucine zipper bZIP transcription factor	--	*E*. *granulosus*
**emu-let-7-5p**	EmuJ_000379600	Nuclear receptor 2DBD gamma	--	*E*. *granulosus* and *T*. *solium*
	EmuJ_000813900	Homeobox protein Hox B4a	--	*E*. *granulosus* and *T*. *solium*
	EmuJ_001053100	Transcription factor sum	--	*E*. *granulosus* and *T*. *solium*
**emu-miR-71-5p**	EmuJ_001059700	Aurora kinase A	Oocyte meiosis	*T*. *solium*
	EmuJ_000646000	Mitotic checkpoint serine:threonine protein	Cell cycle	*E*. *granulosus*
	EmuJ_000456500	G1:S specific cyclin D1	Cell cycle	*E*. *granulosus* and *T*. *solium*
	EmuJ_000974400	Cyclin G2	p53 signaling pathway	*E*. *granulosus*
	EmuJ_000866400	Polycomb group RING finger protein 2	Signaling pathways regulating pluripotency of stem cells	*E*. *granulosus* and *T*. *solium*
	EmuJ_001141100	Regulatory associated protein of mTOR	mTOR signaling pathway	*E*. *granulosus*
	EmuJ_000468300	Target of rapamycin complex 2 subunit MAPKAP1	mTOR signaling pathway	*E*. *granulosus* and *T*. *solium*
	EmuJ_000445850	Mitochondrial import inner membrane translocase subunit Tim23 putative	Longevity regulating pathway - worm	*T*. *solium*

^a^ Gene Description according to WBPS14 (WS271).

MiR-10 is a deeply conserved miRNA known by its role in development and hox regulation. Among the selected targets for emu-miR-10, we found a Mitogen activated protein kinase kinase kinase from the MAPK signaling pathway. Also, the gene pangolin J from the canonical Wnt signaling pathway had a target site for miR-10. In addition, one homeobox protein from the Meis family may be regulated by emu-miR-10. These three targets were conserved in *E*. *granulosus* and *T*. *solium*.

MiR-1 is a highly conserved miRNA known for its role in muscle development. Among the selected targets for emu-miR-1 we found genes encoding cytoskeleton proteins such as light and heavy dyneins which were conserved in *E*. *granulosus*. Also, we found the gene Ezrin-like protein (Elp) that is involved in the regulation of actin cytoskeleton as a putative target of emu-miR-1 conserved in *T*. *solium*. This result agrees with the high expression of emu-miR-1 in *E*. *multilocularis* protoscoleces. In addition, we found an Activin gene from the TGF-beta signaling pathway, which was conserved in *E*. *granulosus*. Interestingly, the Activin gene is involved in immunomodulation in *E*. *multilocularis* [101].

MiR-9 is a deeply conserved miRNA across evolution known to be involved in neural development. Among the selected targets for emu-miR-9 we found a gene encoding a carbonic anhydrase, which is the ortholog of *C*. *elegans* cah-1 that is expressed in different neurons and head ganglion. Another target of emu-miR-9 was the gene Peregrin, a putative histone modification protein. Both targets were conserved in *E*. *granulosus*.

MiR-4989 is a divergent member of the miR-277 family. This protostomian-specific family is known to be involved in amino acid catabolism in *Drosophila*. Recently, miR-4989 was shown to be involved in development of juvenile worms in *S*. *mansoni* [47]. Among the selected targets for emu-miR-4989 we found a general transcription factor IIH subunit that mapped to basal transcription factors and a basic leucine zipper bZIP transcription factor that were conserved in *E*. *granulosus*.

Let-7 is a bilaterian miRNA, highly divergent in *Echinococcus*, known to be essential for temporal development in *C*. *elegans*. Among the selected targets for emu-let-7 we found a nuclear receptor that may be potentially involved in transcription regulation and a homeobox protein, which is the ortholog of *C*.*elegans* lin-39, a homeodomain protein involved in cell fate specification, regulation of transcription and development. Also, we found that transcription factor sum, the ortholog of *C*. *elegans* hlh-1, a transcription factor required during embryogenesis for proper bodywall muscle development and function, had a miRNA target site for emu-let-7. The three selected targets were conserved in *E*. *granulosus* and *T*. *solium*.

MiR-71 is bilaterian miRNA absent in vertebrates that is known to be involved in promotion of longevity and neuronal asymmetry in *C*. *elegans*. Among selected targets of emu-miR-71, we found genes that may be involved in cell cycle in *E*. *multilocularis* such as an Aurora kinase A, a mitotic checkpoint serine:threonine protein and cyclins D1 and G2 that mapped to different pathways related to cell growth and death. Interestingly, we found a potential target of emu-miR-71, Polycomb group RING finger protein 2 that mapped to signaling pathways regulating pluripotency of stem cells, conserved in both closely related species *E*. *granulosus* and *T*. *solium*. These results are in coincidence with the expression of emu-miR-71 in *germinative* cells found in this work. Other relevant targets of miR-71 that may be involved in regulation of lifespan and aging were a Regulatory associated protein of mTOR, Target of rapamycin complex 2 subunit MAPKAP1 and a Mitochondrial import inner membrane translocase subunit that mapped to mTOR signaling pathway or longevity regulating pathway. Evolutionary conservation of *E*. *multilocularis* miR-71 targets in *E*. *granulosus* and *T*. *solium* is shown in Table 2.

We recently reported 16 targets of emu-miR-71 in *E*. *multilocularis* PC1 cells with inverse expression between the miRNA and the target after knocking down miR-71 [14]. Here, we broaden the repertoire of putative targets of emu-miR-71 by performing a bioinformatic approach based on a different input set of 3’UTRs for miRNA target prediction that varied not only in the total number of 3’UTRs, but also in their length. We determined that eight out of 16 targets of emu-miR-71 that increased their expression after knocking down this miRNA in *E*. *multilocularis* PC1 cells from our previous study [14] overlapped with emu*-*miR-71 targets predicted here (S5 Table). This set from overlapped targets, that includes genes with important functions for parasite development and survival in the host, such as EmTip [64] and frizzled [92], provides a shortened list of targets to be further experimentally validated and analyzed in the future. From the eight target genes that did not overlap with the targets from this study, five had 3’ UTRs that were absent from the set of 6,227 3’UTRs used as input for miRNA target prediction in the present study, whereas the remaining three had 3’UTRs of a different length.

Taken together, *in silico* functional analyses of predicted miRNA targets suggest conserved roles for miR-10, miR-1, miR-9, miR-4989, let-7 and miR-71 the most highly expressed miRNAs in all *E*. *multilocularis* samples analyzed. In addition, the potential roles of predicted targets in *E*. *multilocularis* reveals that these miRNAs may be involved in essential processes for survival and development in the host suggesting the importance of the miRNA post-transcriptional regulation mechanism in the biology of the parasite. In this study, we provide some potential miRNA-target interactions to guide future experimental functional studies in *E*. *multilocularis* that might lead to improved treatment and control of this neglected parasite, since miRNAs that are involved in key biological processes may provide novel therapeutic targets.

In this work, we analyzed for the first time the expression profile of *E*. *multilocularis* miRNAs throughout metacestode development *in vitro*. We found that a small number of miRNAs are highly expressed throughout the life cycle stages or *in vitro* conditios analyzed, whereas most of them are weakly expressed. We predicted functional roles for highly expressed miRNAs and found that they could be involved in essential roles for survival and development in the host. We also determined for the first time the expression of miR-71, a highly expressed miRNA that is absent in the human host, in germinative cells of the germinal layer in *E*. *multilocularis* metacestodes cultured *in vitro*. Germinative cells are a relevant cell type to target for anti-echinococcosis drug development. In this study, we provide a resource to guide future miRNA functional analyses in *E*. *multilocularis*. MiRNAs involved in parasite essential functions, highly expressed and/or expressed in germinative cells in the metacestode stage and absent in the host, such as miR-71 and miR-4989, may represent selective therapeutic targets for treatment and control of AE.

## Supporting information

S1 Fig Experimental and bioinformatic workflow for miRNA expression profiling and differential expression analysis. MCvivo: metacestodes extracted from experimentally infected jirds, MCvitro: metacestodes cultured *in vitro* in aerobic conditions, MCana: metacestodes cultured *in vitro* in axenic and anaerobic conditions, PC1: primary cells cultured *in vitro* for 48 hs, PC2: primary cells cultured *in vitro* for 7 days containing central cavities and PC3: primary cells cultured *in vitro* for 21 days containing several central cavities and released mini vesicles (metacestodes), naPS: non-activated protoscoleces, aPS: activated protoscoleces.
(TIF)

S2 Fig Principal component analysis of the miRNA expression data from different *Echinococcus multilocularis* samples. PCA plot of all biological replicates from different *E*. *multilocularis* samples based on normalized miRNA read counts. Each dot indicates one biological replicate from a given sample. The dot corresponding to the biological replicate aPS_1 is indicated. MCvivo: metacestodes extracted from experimentally infected jirds, MCvitro: metacestodes cultured *in vitro* in aerobic conditions, MCana: metacestodes cultured *in vitro* in axenic and anaerobic conditions, naPS: non-activated protoscoleces, aPS: activated protoscoleces, PC1: primary cell cultured *in vitro* for 48 hs, PC2: primary cells cultured *in vitro* for 7 days and PC3: primary cells cultured *in vitro* for 21 days.
(TIF)

S3 Fig Scatter plots showing correlation of microRNA expression between pairs of biological replicates from different *Echinococcus multilocularis* samples. Pearson’s correlation coefficients are shown for each pair of biological replicates in the upper panels. MCvivo (A). MCvitro (B). MCana (C). PC1 (D). PC2 (E). PC3 (F). naPS (G). aPS (H).
(TIF)

S4 Fig Scatter plot showing correlation of microRNA expression between pairs of biological replicates from *Echinococcus multilocularis* PC2 and PC3 samples. Pearson’s correlation coefficients are shown for each pair of biological replicates in the upper panels.
(TIF)

S5 Fig Whole-mount *in situ* hybridization (WMISH) of miR-71 and a scrambled LNA probes in *Echinococcus multilocularis* metacestodes cultured *in vitro* (MCvitro). Germinal layer with a brood capsule showing nuclear labeling with DAPI (blue signal) and scrambled LNA probe as negative control (no signal) (A). Germinal layer with a brood capsule showing nuclear labeling with DAPI (blue signal) and miR-71 expression (green signal) (B).
(TIF)

S1 Table General results of sequenced small RNA libraries from *Echinococcus multilocularis*. (DOCX)

S2 Table *Echinococcus multilocularis* microRNA (miRNA) sequences identified in this study and their genome location. (XLSX)

S3 Table *Echinococcus multilocularis* microRNA (miRNA) clusters identified in this study. (XLSX)

S4 Table Normalized expression profiles of the whole repertoire of the *Echinococcus multilocularis* mature *microRNAs (miRNAs)* identified in this study. (XLSX)

S5 Table All predicted target sites of the most expressed microRNAs (miRNAs) in *Echinococcus multilocularis* using miRanda algorithm. (XLSX)

S6 Table All predicted target sites of the most expressed microRNAs (miRNAs) in *Echinococcus multilocularis* using RNAhybrid algorithm. (XLSX)

S7 Table Evolutionary conservation analysis of *Echinococcus multilocularis* microRNA (miRNA) target sites predicted by both miRanda and RNAhybrid algorithms in the related species *Echinococcus granulosus* and *Taenia solium*. (XLSX)

S8 Table Gene ontology (GO) enrichment analysis of microRNA (miRNA) predicted targets in *Echinococcus multilocularis*. (XLSX)

S9 Table Predicted target genes of *Echinococcus multilocularis* most expressed microRNAs (miRNAs) mapping to KEGG pathways. (XLSX)

S1 File Video of whole-mount *in situ* hybridization (WMISH) of miR-71 in *Echinococcus multilocularis* metacestodes cultured *in vitro* (MCvitro). Germinal layer showing proliferating cells with EdU (magenta signal) with and without expression of miR-71 (green signal).
(AVI)

S2 File Database of *Echinococcus multilocularis* rRNAs, tRNAs, mRNAs, snRNAs, snoRNAs and miRNAs. All mapped reads were BLAST searched (e-value 0.01, short sequences optimization) against this in-house database for classification into categories.
(FA) Click here for additional data file.

## References

[1] Ito A. Review of “Echinococcus and Echinococcosis, Part A.” edited by Andrew Thompson R. C., Lymbery Alan J. and Peter Deplazes. Parasit Vectors. 2017;10: 408. 10.1186/s13071-017-2345-8 PMC5583985 28870244

[2] Eckert J, Deplazes P. Biological, Epidemiological, and Clinical Aspects of Echinococcosis, a Zoonosis of Increasing Concern. Clinical Microbiology Reviews. 2004. pp. 107–135. 10.1128/cmr.17.1.107-135.2004 14726458 PMC321468

[3] Koziol U, Rauschendorfer T, Zanon Rodríguez L, Krohne G, Brehm K. The unique stem cell sysem of the immortal larva of the human parasite Echinococcus multilocularis. Evodevo. 2014;5: 10. 10.1186/2041-9139-5-10 24602211 PMC4015340

[4] Thompson RCA. Biology and Systematics of Echinococcus. Advances in parasitology. 2017. pp. 65–109. 10.1016/bs.apar.2016.07.001 28131366

[5] Brunetti E, Kern P, Vuitton DA, Writing Panel for the WHO-IWGE. Expert consensus for the diagnosis and treatment of cystic and alveolar echinococcosis in humans. Acta Tropica Apr, 2010 pp. 1–16. 10.1016/j.actatropica.2009.11.001 19931502

[6] Kern P. Clinical features and treatment of alveolar echinococcosis. Curr Opin Infect Dis. 2010;23: 505–512. 10.1097/QCO.0b013e32833d7516 20683265

[7] Casulli A. Recognising the substantial burden of neglected pandemics cystic and alveolar echinococcosis. The Lancet Global Health. Elsevier Ltd; 2020. pp. e470–e471. 10.1016/S2214-109X(20)30066-8 32199112

[8] Lundström-Stadelmann B, Rufener R, Ritler D, Zurbriggen R, Hemphill A. The importance of being parasiticidal… an update on drug development for the treatment of alveolar echinococcosis. Food Waterborne Parasitol. 2019;15. 10.1016/j.fawpar.2019.e00040 32095613 PMC7034016

[9] Brehm K, Koziol U. On the importance of targeting parasite stem cells in anti-echinococcosis drug development. Parasite. 2014;21: 72. 10.1051/parasite/2014070 25526547 PMC4271656

[10] Spiliotis M, Lechner S, Tappe D, Scheller C, Krohne G, Brehm K. Transient transfection of Echinococcus multilocularis primary cells and complete in vitro regeneration of metacestode vesicles. Int J Parasitol. 2008;38: 1025–1039. 10.1016/j.ijpara.2007.11.002 18086473

[11] Spiliotis M, Brehm K. Axenic in vitro cultivation of Echinococcus multilocularis metacestode vesicles and the generation of primary cell cultures. Methods Mol Biol. 2009;470: 245–262. 10.1007/978-1-59745-204-5_17 19089387

[12] Hemer S, Konrad C, Spiliotis M, Koziol U, Schaack D, Förster S, et al. Host insulin stimulates Echinococcus multilocularis insulin signalling pathways and larval development. BMC Biol. 2014;12: 5. 10.1186/1741-7007-12-5 24468049 PMC3923246

[13] Förster S, Koziol U, Schäfer T, Duvoisin R, Cailliau K, Vanderstraete M, et al. The role of fibroblast growth factor signalling in Echinococcus multilocularis development and host-parasite interaction. Siles-Lucas M, editor. PLoS Negl Trop Dis. 2019;13: e0006959. 10.1371/journal.pntd.0006959 30849083 PMC6426264

[14] Pérez MG, Spiliotis M, Rego N, Macchiaroli N, Kamenetzky L, Holroyd N, et al. Deciphering the role of miR-71 in Echinococcus multilocularis early development in vitro. PLoS Negl Trop Dis. 2019;13: e0007932. 10.1371/journal.pntd.0007932 31881019 PMC6957206

[15] Bartel DP. Metazoan MicroRNAs. Cell. 2018;173: 20–51. 10.1016/j.cell.2018.03.006 29570994 PMC6091663

[16] Bartel DP. MicroRNAs: Genomics, Biogenesis, Mechanism, and Function. Cell. 2004;116: 281–297. 10.1016/s0092-8674(04)00045-5 14744438

[17] Bartel DP. MicroRNAs: target recognition and regulatory functions. Cell. 2009;136: 215–233. 10.1016/j.cell.2009.01.002 19167326 PMC3794896

[18] Cai P, Gobert GN, McManus DP. MicroRNAs in Parasitic Helminthiases: Current Status and Future Perspectives. Trends Parasitol. 2016;32: 71–86. 10.1016/j.pt.2015.09.003 26489492

[19] Gutierrez-Loli R, Orrego MA, Sevillano-Quispe OG, Herrera-Arrasco L, Guerra-Giraldez C. MicroRNAs in Taenia solium neurocysticercosis: Insights as promising agents in host-parasite interaction and their potential as biomarkers. Frontiers in Microbiology. Frontiers Media S.A.; 2017. 10.3389/fmicb.2017.01905 PMC5626859 29033926

[20] Alizadeh Z, Mahami-Oskouei M, Spotin A, Kazemi T, Ahmadpour E, Cai P, et al. Parasite-derived microRNAs in plasma as novel promising biomarkers for the early detection of hydatid cyst infection and post-surgery follow-up. Acta Trop. 2020;202: 105255. 10.1016/j.actatropica.2019.105255 31682814

[21] Ancarola ME, Marcilla A, Herz M, Macchiaroli N, Pérez M, Asurmendi S, et al. Cestode parasites release extracellular vesicles with microRNAs and immunodiagnostic proteins cargo. Int J Parasitol. 2017;47: 675–686. 10.1016/j.ijpara.2017.05.003 28668323

[22] Samoil V, Dagenais M, Ganapathy V, Aldridge J, Glebov A, Jardim A, et al. Vesicle-based secretion in schistosomes: Analysis of protein and microRNA (miRNA) content of exosome-like vesicles derived from Schistosoma mansoni. Sci Rep. 2018;8: 3286. 10.1038/s41598-018-21587-4 29459722 PMC5818524

[23] Quintana JF, Kumar S, Ivens A, Chow FWN, Hoy AM, Fulton A, et al. Comparative analysis of small RNAs released by the filarial nematode Litomosoides sigmodontis in vitro and in vivo. Bennuru S, editor. PLoS Negl Trop Dis. 2019;13: e0007811. 10.1371/journal.pntd.0007811 31770367 PMC6903752

[24] Sotillo J, Robinson MW, Kimber MJ, Cucher M, Ancarola ME, Nejsum P, et al. The protein and microRNA cargo of extracellular vesicles from parasitic helminths–current status and research priorities. Int J Parasitol. 2020;50: 635–645. 10.1016/j.ijpara.2020.04.010 32652128

[25] Ancarola ME, Lichtenstein G, Herbig J, Holroyd N, Mariconti M, Brunetti E, et al. Extracellular non-coding RNA signatures of the metacestode stage of Echinococcus multilocularis. PLoS Negl Trop Dis. 2020;14: e0008890. 10.1371/journal.pntd.0008890 33253209 PMC7728270

[26] Manzano-Román R, Siles-Lucas M. MicroRNAs in parasitic diseases: potential for diagnosis and targeting. Mol Biochem Parasitol. 2012;186: 81–6. 10.1016/j.molbiopara.2012.10.001 23069113

[27] Britton C, Winter AD, Gillan V, Devaney E. MicroRNAs of parasitic helminths—Identification, characterization and potential as drug targets. Int J Parasitol Drugs Drug Resist. 2014;4: 85–94. 10.1016/j.ijpddr.2014.03.001 25057458 PMC4095049

[28] Arora N, Tripathi S, Singh AK, Mondal P, Mishra A, Prasad A. Micromanagement of immune system: Role of miRNAs in helminthic infections. Frontiers in Microbiology. Frontiers Media S.A.; 2017. 10.3389/fmicb.2017.00586 28450853 PMC5390025

[29] Cucher M, Macchiaroli N, Kamenetzky L, Maldonado L, Brehm K, Rosenzvit MC. High-throughput characterization of Echinococcus spp. metacestode miRNomes. Int J Parasitol. 2015;45: 253–267. 10.1016/j.ijpara.2014.12.003 25659494

[30] Macchiaroli N, Cucher M, Zarowiecki M, Maldonado L, Kamenetzky L, Rosenzvit MC. microRNA profiling in the zoonotic parasite Echinococcus canadensis using a high-throughput approach. Parasit Vectors. 2015;8: 83. 10.1186/s13071-015-0686-8 25656283 PMC4326209

[31] Basika T, Macchiaroli N, Cucher M, Espínola S, Kamenetzky L, Zaha A, et al. Identification and profiling of microRNAs in two developmental stages of the model cestode parasite Mesocestoides corti. Mol Biochem Parasitol. 2016;210: 37–49. 10.1016/j.molbiopara.2016.08.004 27544036

[32] Pérez MG, Macchiaroli N, Lichtenstein G, Conti G, Asurmendi SS, Milone DH, et al. microRNA analysis of Taenia crassiceps cysticerci under praziquantel treatment and genome-wide identification of Taenia solium miRNAs. Int J Parasitol. 2017;47: 643–653. 10.1016/j.ijpara.2017.04.002 28526608

[33] Macchiaroli N, Cucher M, Kamenetzky L, Yones C, Bugnon L, Berriman M, et al. Identification and expression profiling of microRNAs in Hymenolepis. Int J Parasitol. 2019;49: 211–223. 10.1016/j.ijpara.2018.07.005 30677390

[34] Brehm K, Wolf M, Beland H, Kroner A, Frosch M. Analysis of differential gene expression in Echinococcus multilocularis larval stages by means of spliced leader differential display. Int J Parasitol. 2003;33: 1145–1159. 10.1016/s0020-7519(03)00169-3 13678631

[35] Gelmedin V, Spiliotis M, Brehm K. Molecular characterisation of MEK1/2- and MKK3/6-like mitogen-activated protein kinase kinases (MAPKK) from the fox tapeworm Echinococcus multilocularis. Int J Parasitol. 2010;40: 555–567. 10.1016/j.ijpara.2009.10.009 19887070

[36] Tsai IJ, Zarowiecki M, Holroyd N, Garciarrubio A, Sánchez-Flores A, Brooks KL, et al. The genomes of four tapeworm species reveal adaptations to parasitism. Nature. 2013;496: 57–63. 10.1038/nature12031 23485966 PMC3964345

[37] Howe KL, Bolt BJ, Shafie M, Kersey P, Berriman M. WormBase ParaSite—a comprehensive resource for helminth genomics. Mol Biochem Parasitol. 2017;215: 2–10. 10.1016/j.molbiopara.2016.11.005 27899279 PMC5486357

[38] Kozomara A, Griffiths-Jones S. MiRBase: Integrating microRNA annotation and deep-sequencing data. Nucleic Acids Res. 2011;39: D152–D157. 10.1093/nar/gkq1027 21037258 PMC3013655

[39] Martin M. Cutadapt removes adapter sequences from high-throughput sequencing reads. EMBnet.journal. 2011;17: 10. 10.14806/ej.17.1.200

[40] Langmead B, Salzberg SL. Fast gapped-read alignment with Bowtie 2. Nat Methods. 2012;9: 357–359. 10.1038/nmeth.1923 22388286 PMC3322381

[41] Altschul SF, Gish W, Miller W, Myers EW, Lipman DJ. Basic local alignment search tool. J Mol Biol. 1990;215: 403–410. 10.1016/S0022-2836(05)80360-2 2231712

[42] Sweeney BA, Petrov AI, Ribas CE, Finn RD, Bateman A, Szymanski M, et al. RNAcentral 2021: Secondary structure integration, improved sequence search and new member databases. Nucleic Acids Res. 2021;49: D212–D220. 10.1093/nar/gkaa921 33106848 PMC7779037

[43] Friedländer MR, Mackowiak SD, Li N, Chen W, Rajewsky N, Friedlander MR, et al. MiRDeep2 accurately identifies known and hundreds of novel microRNA genes in seven animal clades. Nucleic Acids Res. 2012;40: 37–52. 10.1093/nar/gkr688 21911355 PMC3245920

[44] Bai Y, Zhang Z, Jin L, Kang H, Zhu Y, Zhang L, et al. Genome-wide sequencing of small RNAs reveals a tissue-specific loss of conserved microRNA families in Echinococcus granulosus. BMC Genomics. 2014;15: 736. 10.1186/1471-2164-15-736 25168356 PMC4156656

[45] de Souza Gomes M, Muniyappa MK, Carvalho SG, Guerra-Sá R, Spillane C, Guerra-Sa R, et al. Genome-wide identification of novel microRNAs and their target genes in the human parasite Schistosoma mansoni. Genomics. 2011;98: 96–111. 10.1016/j.ygeno.2011.05.007 21640815

[46] Marco A, Kozomara A, Hui JHL, Emery AM, Rollinson D, Griffiths-Jones S, et al. Sex-Biased Expression of MicroRNAs in Schistosoma mansoni. Jones MK, editor. PLoS Negl Trop Dis. 2013;7: e2402. 10.1371/journal.pntd.0002402 24069470 PMC3772069

[47] Protasio A V, van Dongen S, Collins J, Quintais L, Ribeiro DM, Sessler F, et al. MiR-277/4989 regulate transcriptional landscape during juvenile to adult transition in the parasitic helminth Schistosoma mansoni. Brindley PJ, editor. PLoS Negl Trop Dis. 2017;11: e0005559. 10.1371/journal.pntd.0005559 28542189 PMC5459504

[48] Fromm B, Worren MM, Hahn C, Hovig E, Bachmann L. Substantial loss of conserved and gain of novel MicroRNA families in flatworms. Mol Biol Evol. 2013;30: 2619–2628. 10.1093/molbev/mst155 24025793 PMC3840308

[49] Friedländer MR, Adamidi C, Han T, Lebedeva S, Isenbarger TA, Hirst M, et al. High-resolution profiling and discovery of planarian small RNAs. Proc Natl Acad Sci U S A. 2009;106: 11546–11551. 10.1073/pnas.0905222106 19564616 PMC2703670

[50] Lu Y, Smielewska M, Palakodeti D, Lovci MT, Aigner S, Yeo GW, et al. Deep sequencing identifies new and regulated microRNAs in Schmidtea mediterranea. RNA. 2009;15: 1483–1491. 10.1261/rna.1702009 19553344 PMC2714757

[51] Griffiths-Jones S, Saini HK, van Dongen S, Enright AJ. miRBase: Tools for microRNA genomics. Nucleic Acids Res. 2008;36: D154—8. 10.1093/nar/gkm952 17991681 PMC2238936

[52] Love MI, Huber W, Anders S. Moderated estimation of fold change and dispersion for RNA-seq data with DESeq2. Genome Biol. 2014;15: 550. 10.1186/s13059-014-0550-8 25516281 PMC4302049

[53] Anders S, Huber W, S A, W H. DESeq: Differential expression analysis for sequence count data. Genome Biol. 2010;11: R106. 10.1186/gb-2010-11-10-r106 20979621 PMC3218662

[54] Schindelin J, Arganda-Carreras I, Frise E, Kaynig V, Longair M, Pietzsch T, et al. Fiji: An open-source platform for biological-image analysis. Nature Methods. Nat Methods; 2012. pp. 676–682. 10.1038/nmeth.2019 22743772 PMC3855844

[55] Macchiaroli N, Maldonado LL, Zarowiecki M, Cucher M, Gismondi MMI, Kamenetzky L, et al. Genome-wide identification of microRNA targets in the neglected disease pathogens of the genus Echinococcus. Mol Biochem Parasitol. 2017;17: 30048–8. 10.1016/j.molbiopara.2017.04.001 28385564

[56] Enright AJ, John B, Gaul U, Tuschl T, Sander C, Marks DS. MicroRNA targets in Drosophila. Genome Biol. 2003;5: 1–14. 10.1186/gb-2003-5-1-r1 14709173 PMC395733

[57] Krüger J, Rehmsmeier M. RNAhybrid: MicroRNA target prediction easy, fast and flexible. Nucleic Acids Res. 2006;34: W451–W454. 10.1093/nar/gkl243 16845047 PMC1538877

[58] Smedley D, Haider S, Durinck S, Pandini L, Provero P, Allen J, et al. The BioMart community portal: An innovative alternative to large, centralized data repositories. Nucleic Acids Res. 2015;43: W589–W598. 10.1093/nar/gkv350 25897122 PMC4489294

[59] Kanehisa M. Toward understanding the origin and evolution of cellular organisms. Protein Science. Blackwell Publishing Ltd; 2019. pp. 1947–1951. 10.1002/pro.3715 PMC6798127 31441146

[60] Kanehisa M, Sato Y, Kawashima M, Furumichi M, Tanabe M. KEGG as a reference resource for gene and protein annotation. Nucleic Acids Res. 2016;44: D457–D462. 10.1093/nar/gkv1070 26476454 PMC4702792

[61] Kanehisa M, Sato Y, Morishima K. BlastKOALA and GhostKOALA: KEGG Tools for Functional Characterization of Genome and Metagenome Sequences. J Mol Biol. 2016;428: 726–731. 10.1016/j.jmb.2015.11.006 26585406

[62] Raudvere U, Kolberg L, Kuzmin I, Arak T, Adler P, Peterson H, et al. G:Profiler: A web server for functional enrichment analysis and conversions of gene lists (2019 update). Nucleic Acids Res. 2019;47: W191–W198. 10.1093/nar/gkz369 31066453 PMC6602461

[63] Koziol U. Evolutionary developmental biology (evo-devo) of cestodes. Exp Parasitol. 2017;180: 84–100. 10.1016/j.exppara.2016.12.004 27939766

[64] Nono JK, Lutz MB, Brehm K. EmTIP, a T-Cell immunomodulatory protein secreted by the tapeworm Echinococcus multilocularis is important for early metacestode development. Dalton JP, editor. PLoS Negl Trop Dis. 2014;8: e2632. 10.1371/journal.pntd.0002632 24392176 PMC3879249

[65] Wang J, Czech B, Crunk A, Wallace A, Mitreva M, Hannon GJ, et al. Deep small RNA sequencing from the nematode Ascaris reveals conservation, functional diversification, and novel developmental profiles. Genome Res. 2011;21: 1462–1477. 10.1101/gr.121426.111 21685128 PMC3166831

[66] de Wit E, Linsen SEV V, Cuppen E, Berezikov E. Repertoire and evolution of miRNA genes in four divergent nematode species. Genome Res. 2009;19: 2064–2074. 10.1101/gr.093781.109 19755563 PMC2775598

[67] Menezes MR, Balzeau J, Hagan JP. 3’ RNA uridylation in epitranscriptomics, gene regulation, and disease. Frontiers in Molecular Biosciences. Frontiers Media S.A.; 2018. 10.3389/fmolb.2018.00061 PMC6053540 30057901

[68] Kim YK, Heo I, Kim VN. Modifications of Small RNAs and their associated proteins. Cell. 2010;143: 703–709. 10.1016/j.cell.2010.11.018 21111232

[69] Wheeler BM, Heimberg AM, Moy VN, Sperling EA, Holstein TW, Heber S, et al. The deep evolution of metazoan microRNAs. Evol Dev. 2009;11: 50–68. 10.1111/j.1525-142X.2008.00302.x 19196333

[70] Tarver JE, Sperling EA, Nailor A, Heimberg AM, Robinson JM, King BL, et al. miRNAs: small genes with big potential in metazoan phylogenetics. Mol Biol Evol. 2013;30: 2369–82. 10.1093/molbev/mst133 23913097

[71] Niwa R, Slack FJ. The evolution of animal microRNA function. Curr Opin Genet Dev. 2007;17: 145–150. 10.1016/j.gde.2007.02.004 17317150

[72] Berezikov E. Evolution of microRNA diversity and regulation in animals. Nat Rev Genet. 2011;12: 846–860. 10.1038/nrg3079 22094948

[73] Cai P, Hou N, Piao X, Liu S, Liu H, Yang F, et al. Profiles of small non-coding RNAs in schistosoma japonicum during development. Jones MK, editor. PLoS Negl Trop Dis. 2011;5: e1256. 10.1371/journal.pntd.0001256 21829742 PMC3149011

[74] Cucher M, Prada L, Mourglia-Ettlin G, Dematteis S, Camicia F, Asurmendi S, et al. Identification of Echinococcus granulosus microRNAs and their expression in different life cycle stages and parasite genotypes. Int J Parasitol. 2011;41: 439–448. 10.1016/j.ijpara.2010.11.010 21219906

[75] Winter AD, Weir W, Hunt M, Berriman M, Gilleard JS, Devaney E, et al. Diversity in parasitic nematode genomes: the microRNAs of Brugia pahangi and Haemonchus contortus are largely novel. BMC Genomics. 2012;13: 4. 10.1186/1471-2164-13-4 22216965 PMC3282659

[76] Cai P, Piao X, Hao L, Liu S, Hou N, Wang H, et al. A Deep Analysis of the Small Non-Coding RNA Population in Schistosoma japonicum Eggs. PLoS One. 2013;8. 10.1371/journal.pone.0064003 23691136 PMC3653858

[77] Zhang X, Zabinsky R, Teng Y, Cui M, Han M. microRNAs play critical roles in the survival and recovery of Caenorhabditis elegans from starvation-induced L1 diapause. Proc Natl Acad Sci. 2011;108: 17997–18002. 10.1073/pnas.1105982108 22011579 PMC3207661

[78] Boulias K, Horvitz HR. The C. elegans MicroRNA mir-71 acts in neurons to promote germline-mediated longevity through regulation of DAF-16/FOXO. Cell Metab. 2012;15: 439–450. 10.1016/j.cmet.2012.02.014 22482727 PMC3344382

[79] Finger F, Ottens F, Springhorn A, Drexel T, Proksch L, Metz S, et al. Olfaction regulates organismal proteostasis and longevity via microRNA-dependent signalling. Nature Metabolism. Nature Research; 2019. pp. 350–359. 10.1038/s42255-019-0033-z 31535080 PMC6751085

[80] Hsieh YW, Chang C, Chuang CF. The MicroRNA mir-71 Inhibits Calcium Signaling by Targeting the TIR-1/Sarm1 Adaptor Protein to Control Stochastic L/R Neuronal Asymmetry in C. elegans. PLoS Genet. 2012;8: e1002864. 10.1371/journal.pgen.1002864 22876200 PMC3410857

[81] Yuva-Aydemir Y, Simkin A, Gascon E, Gao F-B. MicroRNA-9: functional evolution of a conserved small regulatory RNA. RNA Biol. 2011;8: 557–64. 10.4161/rna.8.4.16019 21697652 PMC3225974

[82] Reinhart BJ, Slack FJ, Basson M, Pasquinelli a E, Bettinger JC, Rougvie a E, et al. The 21-nucleotide let-7 RNA regulates developmental timing in Caenorhabditis elegans. Nature. 2000;403: 901–906. 10.1038/35002607 10706289

[83] Lund AH. miR-10 in development and cancer. Cell Death Differ. 2010;17: 209–14. 10.1038/cdd.2009.58 19461655

[84] Tehler D, Høyland-Kroghsbo NM, Lund AH. The miR-10 microRNA precursor family. RNA Biol. 2011;8: 728–734. 10.4161/rna.8.5.16324 21881411 PMC3256350

[85] Esslinger SM, Schwalb B, Helfer S, Michalik KM, Witte H, Maier KC, et al. Drosophila miR-277 controls branched-chain amino acid catabolism and affects lifespan. RNA Biol. 2013;10: 1042–1056. 10.4161/rna.24810 23669073 PMC3904584

[86] Sokol NS. Small temporal RNAs in animal development. Curr Opin Genet Dev. 2012;22: 368–373. 10.1016/j.gde.2012.04.001 22578317 PMC3419770

[87] Koziol U, Krohne G, Brehm K. Anatomy and development of the larval nervous system in Echinococcus multilocularis. Front Zool. 2013;10. 10.1186/1742-9994-10-24 23642192 PMC3658878

[88] Camicia F, Herz M, Prada LC, Kamenetzky L, Simonetta SH, Cucher MA, et al. The nervous and prenervous roles of serotonin in Echinococcus spp. Int J Parasitol. 2013;43: 647–659. 10.1016/j.ijpara.2013.03.006 23639266

[89] Ryazansky SS, Gvozdev VA, Berezikov E. Evidence for post-transcriptional regulation of clustered microRNAs in Drosophila. BMC Genomics. 2011;12. 10.1186/1471-2164-12-371 21771325 PMC3150300

[90] Sasidharan V, Lu Y-C, Bansal D, Dasari P, Poduval D, Seshasayee A, et al. Identification of neoblast- and regeneration-specific miRNAs in the planarian Schmidtea mediterranea. RNA. 2013;19: 1394–1404. 10.1261/rna.038653.113 23974438 PMC3854530

[91] Sakamoto T, Sugimura M. [Studies on echinococcosis. 23. Electron microscopical observations on histogenesis of larval Echinococcus multilocularis]. Jpn J Vet Res. 1970;18: 131–44. Available: http://www.ncbi.nlm.nih.gov/pubmed/5313479 5313479

[92] Koziol U, Jarero F, Olson PD, Brehm K. Comparative analysis of Wnt expression identifies a highly conserved developmental transition in flatworms. BMC Biol. 2016;14: 10. 10.1186/s12915-016-0233-x 26941070 PMC4778295

[93] Jiang J, Ge X, Li Z, Wang Y, Song Q, Stanley DW, et al. MicroRNA-281 regulates the expression of ecdysone receptor (EcR) isoform B in the silkworm, bombyx mori. Insect Biochem Mol Biol. 2013;43: 692–700. 10.1016/j.ibmb.2013.05.002 23707601

[94] Cao X, Pfaff SL, Gage FH. A functional study of miR-124 in the developing neural tube. Genes Dev. 2007;21: 531–536. 10.1101/gad.1519207 17344415 PMC1820895

[95] Dang Z, Yagi K, Oku Y, Kouguchi H, Kajino K, Watanabe J, et al. Evaluation of Echinococcus multilocularis tetraspanins as vaccine candidates against primary alveolar echinococcosis. Vaccine. 2009;27: 7339–7345. 10.1016/j.vaccine.2009.09.045 19782112

[96] Gottstein B, Hemphill A. Echinococcus multilocularis: The parasite-host interplay. Exp Parasitol. 2008;119: 447–452. 10.1016/j.exppara.2008.03.002 18410929

[97] Liu J, Zhu L, Wang J, Qiu L, Chen Y, Davis RE, et al. Schistosoma japonicum extracellular vesicle miRNA cargo regulates host macrophage functions facilitating parasitism. Loukas A, editor. PLOS Pathog. 2019;15: e1007817. 10.1371/journal.ppat.1007817 31163079 PMC6548406

[98] Nielsen BS. MicroRNA in situ hybridization. Methods Mol Biol. 2012;822: 67–84. 10.1007/978-1-61779-427-8_5 22144192

[99] Guo X, Zhang X, Yang J, Jin X, Ding J, Xiang H, et al. Suppression of nemo-like kinase by miR-71 in Echinococcus multilocularis. Exp Parasitol. 2017;183: 1–5. 10.1016/j.exppara.2017.10.004 29037783

[100] Zhu L, Zhao J, Wang J, Hu C, Peng J, Luo R, et al. MicroRNAs Are Involved in the Regulation of Ovary Development in the Pathogenic Blood Fluke Schistosoma japonicum. Collins JJ, editor. PLOS Pathog. 2016;12: e1005423. 10.1371/journal.ppat.1005423 26871705 PMC4752461

[101] Li N, Long B, Han W, Yuan S, Wang K. MicroRNAs: Important regulators of stem cells. Stem Cell Research and Therapy. BioMed Central Ltd.; 2017. 10.1186/s13287-017-0551-0 PMC5426004 28494789

[102] Palevich N, Britton C, Kamenetzky L, Mitreva M, de Moraes Mourão M, Bennuru S, et al. Tackling Hypotheticals in Helminth Genomes. Trends in Parasitology. Elsevier Ltd; 2018. pp. 179–183. 10.1016/j.pt.2017.11.007 PMC11021132 29249363

