# Supplementary figures and images for: Expression profiling of Echinococcus multilocularis miRNAs throughout metacestode development in vitro
Source: PLoS Negl Trop Dis. 2021 Mar 22;15(3):e0009297. doi: 10.1371/journal.pntd.0009297 (PMC8016320; doi:10.1371/journal.pntd.0009297)

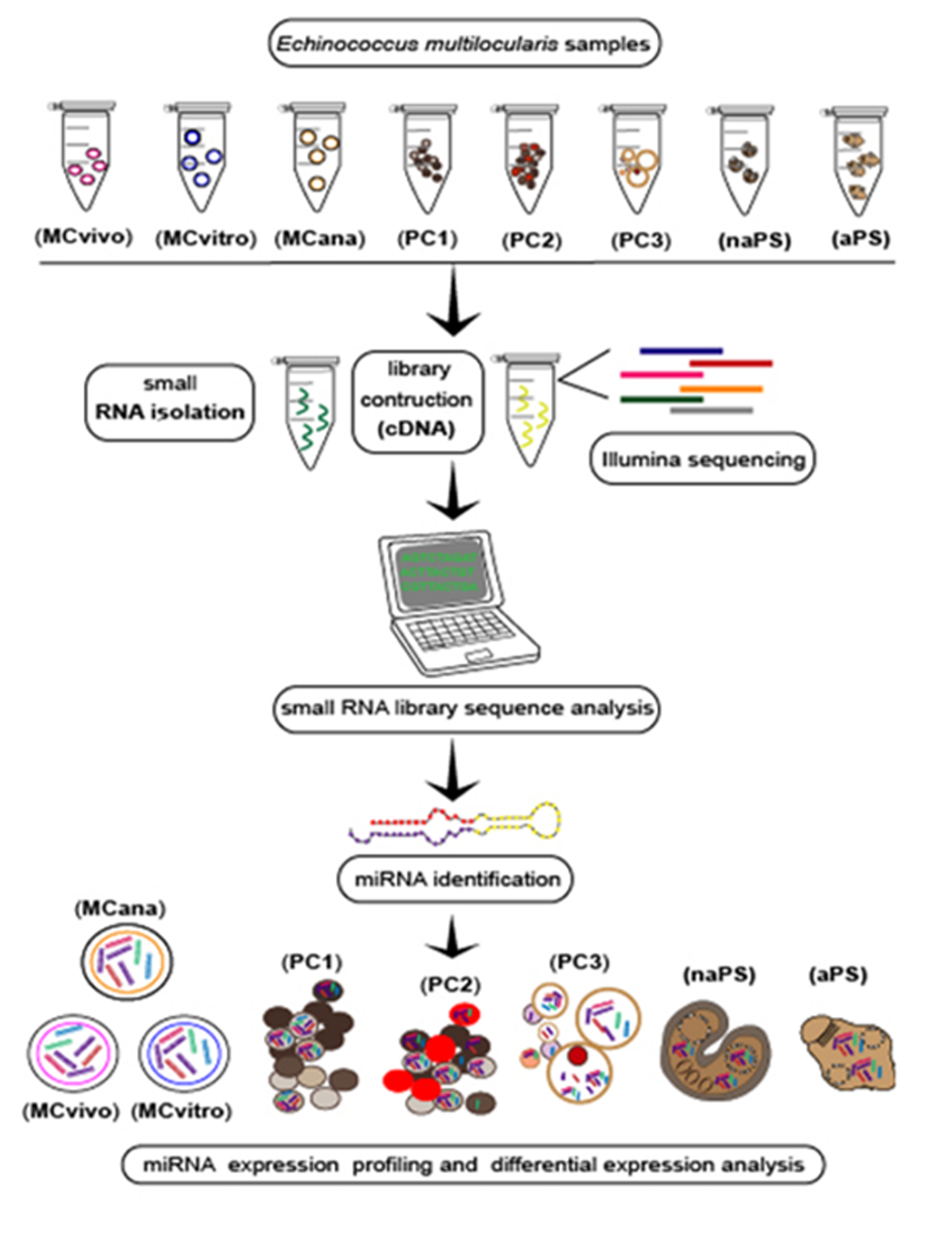

Supplement: S1 Fig — MCvivo: metacestodes extracted from experimentally infected jirds, MCvitro: metacestodes cultured in vitro in aerobic conditions, MCana: metacestodes cultured in vitro in axenic and anaerobic conditions, PC1: primary cells cultured in vitro for 48 hs, PC2: primary cells cultured in vitro for 7 days containing central cavities and PC3: primary cells cultured in vitro for 21 days containing several central cavities and released mini vesicles (metacestodes), naPS: non-activated protoscoleces, aPS: activated protoscoleces. (TIF) [file pntd.0009297.s001.tif]

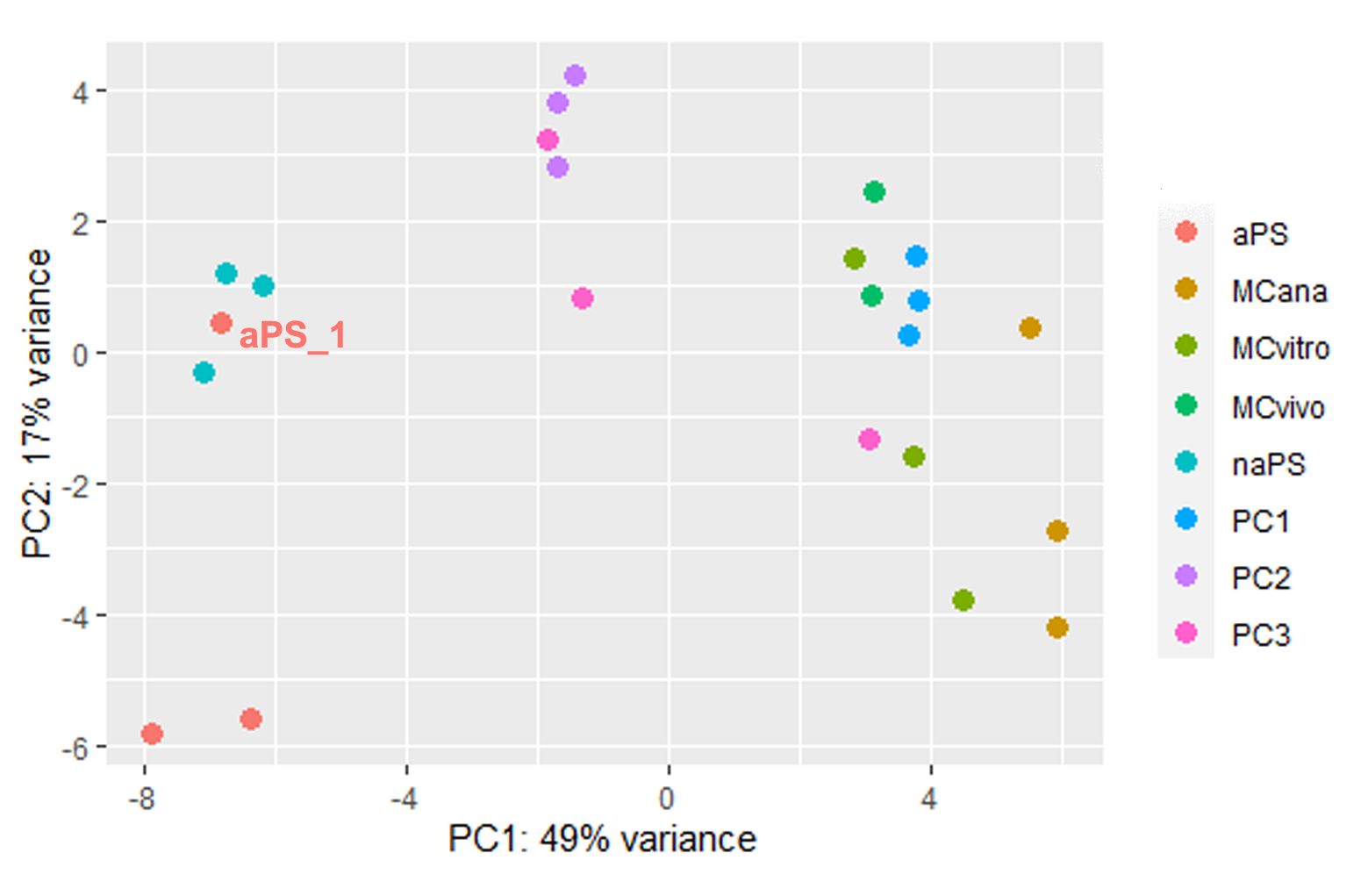

Supplement: S2 Fig — PCA plot of all biological replicates from different E. multilocularis samples based on normalized miRNA read counts. Each dot indicates one biological replicate from a given sample. The dot corresponding to the biological replicate aPS_1 is indicated. MCvivo: metacestodes extracted from experimentally infected jirds, MCvitro: metacestodes cultured in vitro in aerobic conditions, MCana: metacestodes cultured in vitro in axenic and anaerobic conditions, naPS: non-activated protoscoleces, aPS: activated protoscoleces, PC1: primary cell cultured in vitro for 48 hs, PC2: primary cells cultured in vitro for 7 days and PC3: primary cells cultured in vitro for 21 days. (TIF) [file pntd.0009297.s002.tif]

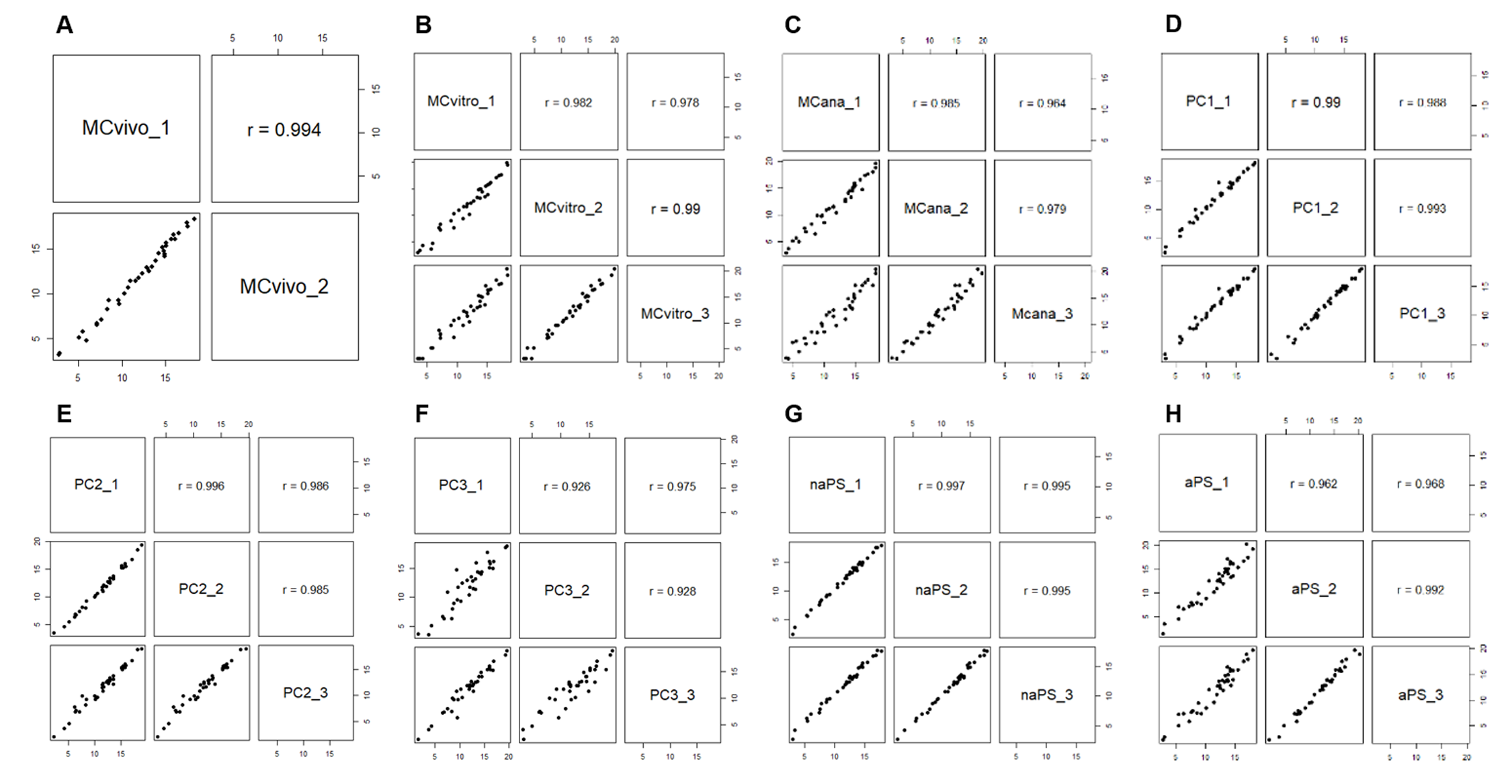

Supplement: S3 Fig — Pearson’s correlation coefficients are shown for each pair of biological replicates in the upper panels. MCvivo (A). MCvitro (B). MCana (C). PC1 (D). PC2 (E). PC3 (F). naPS (G). aPS (H). (TIF) [file pntd.0009297.s003.tif]

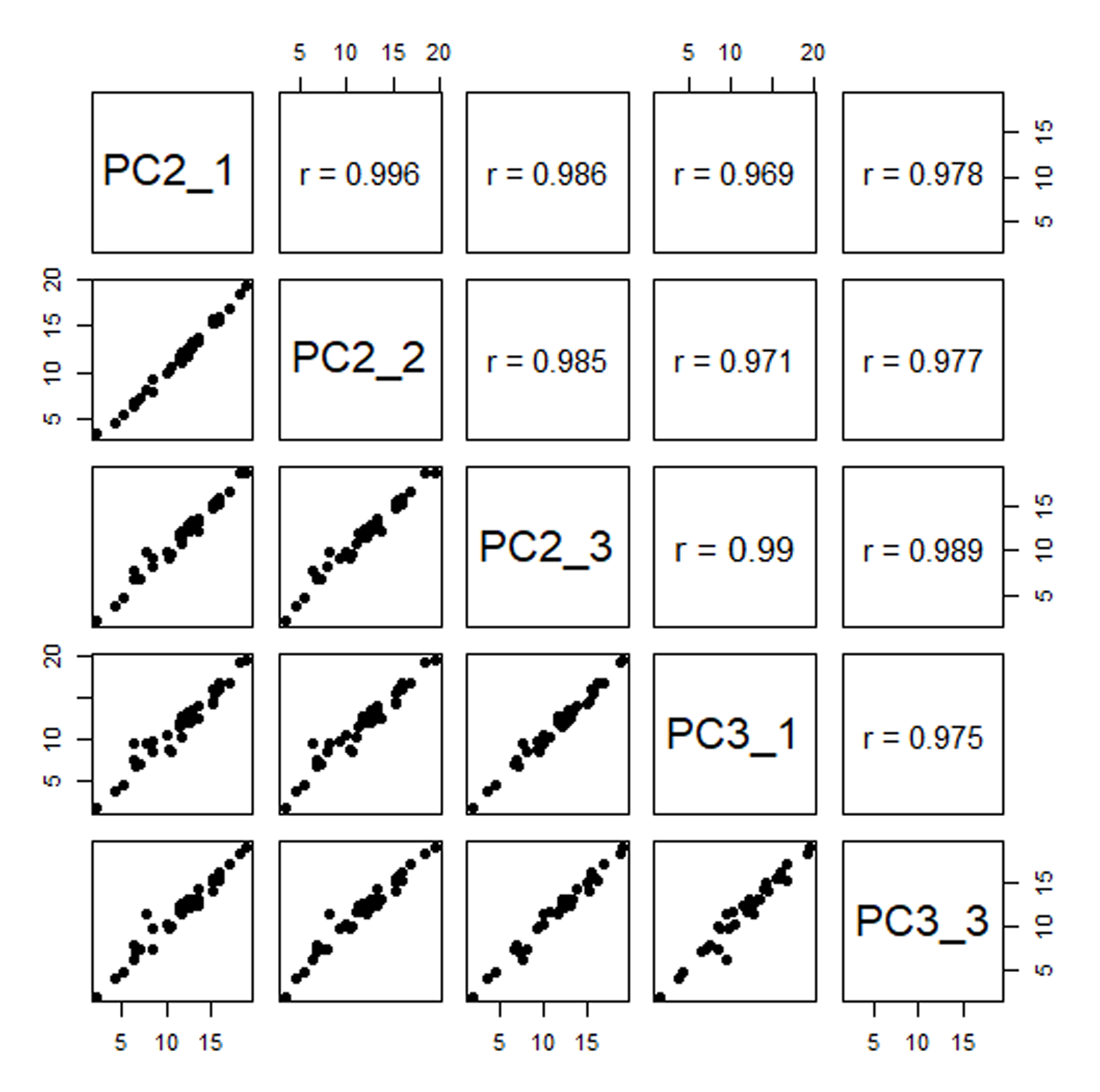

Supplement: S4 Fig — Pearson’s correlation coefficients are shown for each pair of biological replicates in the upper panels. (TIF) [file pntd.0009297.s004.tif]

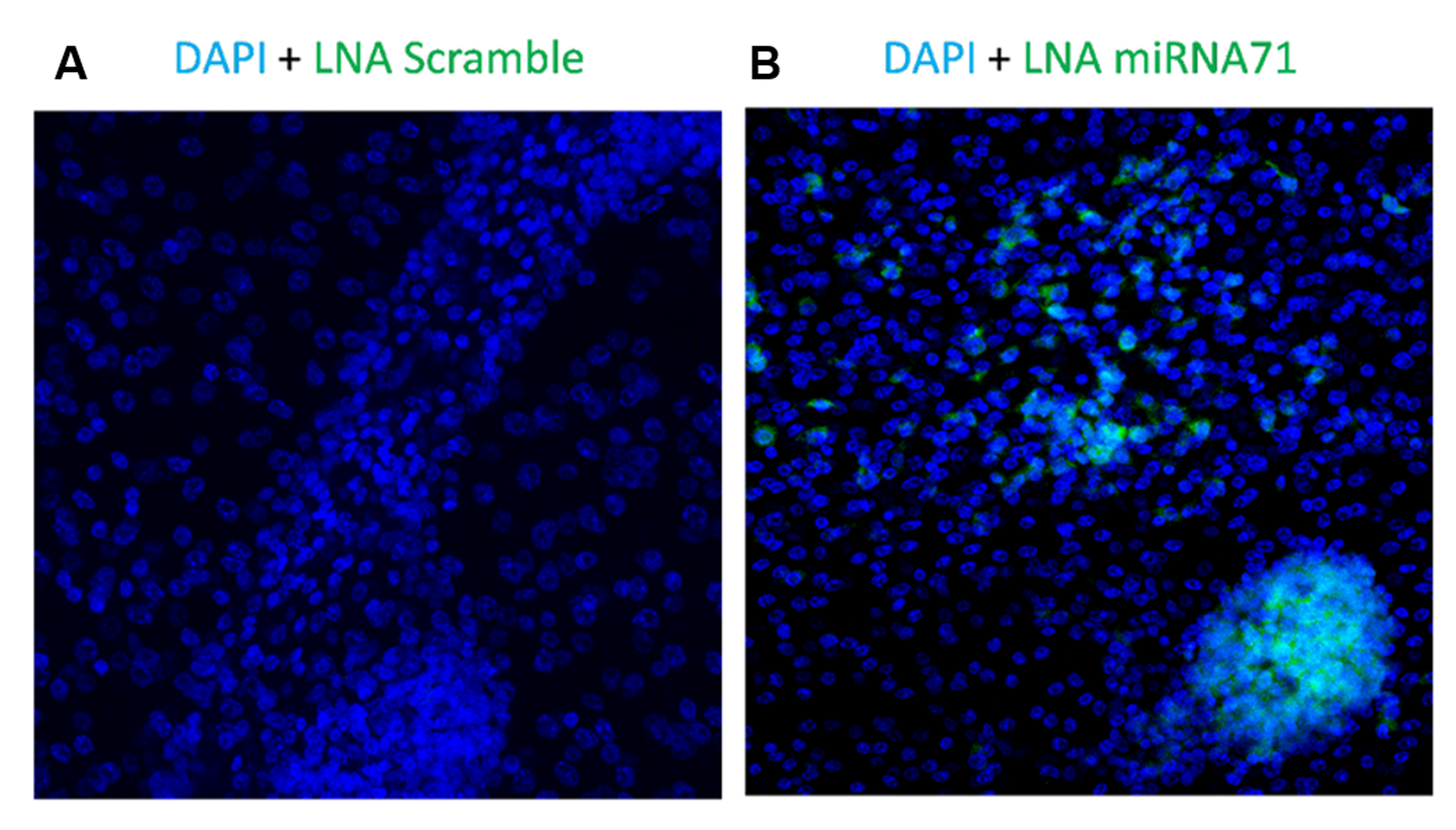

Supplement: S5 Fig — Germinal layer with a brood capsule showing nuclear labeling with DAPI (blue signal) and scrambled LNA probe as negative control (no signal) (A). Germinal layer with a brood capsule showing nuclear labeling with DAPI (blue signal) and miR-71 expression (green signal) (B). (TIF) [file pntd.0009297.s005.tif]
