# Supplementary material for: Expression profiling of Echinococcus multilocularis miRNAs throughout metacestode development in vitro
Source: PLoS Negl Trop Dis. 2021 Mar 22;15(3):e0009297. doi: 10.1371/journal.pntd.0009297 (PMC8016320; doi:10.1371/journal.pntd.0009297)
Supplement: S1 Table — (DOCX) [file pntd.0009297.s006.docx]

**S1 Table**. **General results of sequenced small RNA libraries from *Echinococcus multilocularis.***

| **Sample type^a^** | **Biological replicate** | **Raw reads** | **Trimmed and filtered reads^b^** | **Number of mapped reads** | **Percentage of mapped reads (%)** | **Percentage of reads (%)** | | | | |
| --- | --- | --- | --- | --- | --- | --- | --- | --- | --- | --- |
|  |  |  |  |  |  | **microRNAs** | **tRNAs** | **rRNAs** | **mRNAs** | **others^c^** |
| **MCvivo*** | **1** | 39,289,843 | 22,936,154 | 13,342,962 | 58.17 | 3.86 | 10.16 | 55.22 | 9.84 | 20.92 |
|  | **2** | 9,632,886 | 4,673,966 | 2,652,728 | 56.75 | 6.12 | 13.97 | 57.38 | 11.83 | 10.70 |
| **MCvitro** | **1** | 9,038,443 | 6,709,652 | 6,287,941 | 93.71 | 27.01 | 7.61 | 42.66 | 15.32 | 7.40 |
|  | **2** | 10,369,081 | 7,098,443 | 6,816,118 | 96.02 | 23.27 | 6.01 | 48.19 | 15.42 | 7.11 |
|  | **3** | 12,190,046 | 7,011,619 | 6,632,536 | 94.59 | 17.87 | 11.45 | 46.38 | 14.30 | 10.00 |
| **MCana** | **1** | 5,149,068 | 3,929,397 | 3,761,127 | 95.71 | 12.00 | 3.49 | 59.91 | 18.36 | 6.24 |
|  | **2** | 13,439,605 | 8,278,477 | 7,932,050 | 95.81 | 12.69 | 6.26 | 58.80 | 13.84 | 8.41 |
|  | **3** | 5,899,291 | 3,296,661 | 3,188,085 | 96.71 | 6.59 | 6.85 | 66.11 | 13.26 | 7.19 |
| **PC1** | **1** | 19,338,896 | 11,575,138 | 11,120,135 | 96.26 | 10.68 | 5.54 | 64.23 | 12.06 | 7.49 |
|  | **2** | 10,799,248 | 7,521,472 | 7,185,017 | 95.52 | 11.30 | 6.66 | 59.13 | 14.28 | 8.63 |
|  | **3** | 4,079,286 | 1,756,968 | 1,662,643 | 94.63 | 16.20 | 9.60 | 51.25 | 13.34 | 9.61 |
| **PC2** | **1** | 36,463,652 | 22,697,236 | 21,500,058 | 94.72 | 34.04 | 9.82 | 35.67 | 10.57 | 9.90 |
|  | **2** | 54,673,485 | 37,214,923 | 34,488,490 | 92.67 | 29.00 | 36.59 | 18.39 | 10.06 | 5.96 |
|  | **3** | 14,160,667 | 8,977,247 | 8,426,175 | 93.86 | 48.38 | 7.49 | 20.67 | 9.89 | 13.57 |
| **PC3** | **1** | 21,986,011 | 15,777,872 | 14,853,746 | 94.14 | 17.49 | 43.98 | 22.12 | 8.47 | 7.94 |
|  | **2** | 7,511,405 | 4,663,339 | 4,253,148 | 91.19 | 4.08 | 9.99 | 66.49 | 13.54 | 5.90 |
|  | **3** | 62,288,073 | 41,644,743 | 38,754,829 | 93.06 | 38.42 | 17.77 | 26.18 | 9.53 | 8.10 |
| **naPS** | **1** | 19,553,309 | 15,121,141 | 14,127,932 | 93.43 | 55.84 | 3.64 | 25.74 | 8.06 | 6.72 |
|  | **2** | 23,251,622 | 15,489,677 | 14,415,226 | 93.06 | 61.17 | 3.53 | 19.14 | 8.06 | 8.10 |
|  | **3** | 28,205,549 | 19,693,255 | 16,427,997 | 83.41 | 42.49 | 5.85 | 31.96 | 9.84 | 9.86 |
| **aPS** | **1** | 16,101,703 | 12,202,889 | 11,727,787 | 96.11 | 56.14 | 2.43 | 27.11 | 7.90 | 6.42 |
|  | **2** | 11,380,201 | 8,972,982 | 8,683,185 | 96.77 | 38.66 | 14.93 | 22.51 | 10.34 | 13.56 |
|  | **3** | 3,834,594 | 2,908,114 | 2,818,933 | 96.93 | 41.86 | 4.19 | 34.07 | 13.42 | 6.46 |

^a^ MCvivo: metacestodes extracted from experimentally infected jirds. MCvitro: metacestodes grown *in vitro* in aerobic conditions. MCana: metacestodes grown *in vitro* in anaerobic conditions. PC1: primary cell cultures grown *in vitro* for 48 hs. PC2: primary cell culture grown *in vitro* for 7 days. PC3: primary cell cultures grown *in vitro* for 21 days. naPS: non-activated protoscoleces. aPS: activated protoscoleces.

^b^ Quality and length filtering.

^c^ Reads that matched to snRNAs, snoRNAs or with no match to any category were grouped into “others” category.

^*^ One biological replicate was discarded due to low mapping (<3%).
